# A large-scale comparative study on peptide encodings for biomedical classification

**DOI:** 10.1093/nargab/lqab039

**Published:** 2021-05-22

**Authors:** Sebastian Spänig, Siba Mohsen, Georges Hattab, Anne-Christin Hauschild, Dominik Heider

**Affiliations:** Data Science in Biomedicine, Department of Mathematics and Computer Science, University of Marburg, Hans-Meerwein-Str. 6, D-35032 Marburg, Germany; Data Science in Biomedicine, Department of Mathematics and Computer Science, University of Marburg, Hans-Meerwein-Str. 6, D-35032 Marburg, Germany; Data Science in Biomedicine, Department of Mathematics and Computer Science, University of Marburg, Hans-Meerwein-Str. 6, D-35032 Marburg, Germany; Data Science in Biomedicine, Department of Mathematics and Computer Science, University of Marburg, Hans-Meerwein-Str. 6, D-35032 Marburg, Germany; Data Science in Biomedicine, Department of Mathematics and Computer Science, University of Marburg, Hans-Meerwein-Str. 6, D-35032 Marburg, Germany

## Abstract

Owing to the great variety of distinct peptide encodings, working on a biomedical classification task at hand is challenging. Researchers have to determine encodings capable to represent underlying patterns as numerical input for the subsequent machine learning. A general guideline is lacking in the literature, thus, we present here the first large-scale comprehensive study to investigate the performance of a wide range of encodings on multiple datasets from different biomedical domains. For the sake of completeness, we added additional sequence- and structure-based encodings. In particular, we collected 50 biomedical datasets and defined a fixed parameter space for 48 encoding groups, leading to a total of 397 700 encoded datasets. Our results demonstrate that none of the encodings are superior for all biomedical domains. Nevertheless, some encodings often outperform others, thus reducing the initial encoding selection substantially. Our work offers researchers to objectively compare novel encodings to the state of the art. Our findings pave the way for a more sophisticated encoding optimization, for example, as part of automated machine learning pipelines. The work presented here is implemented as a large-scale, end-to-end workflow designed for easy reproducibility and extensibility. All standardized datasets and results are available for download to comply with FAIR standards.

## INTRODUCTION

With the increasing popularity of machine learning methods, scientists began to use them for a wide range of biomedical applications. A particular application is the prediction of amino acid (AA) sequence properties, for example, a peptide’s antimicrobial efficiency (1), cell-penetrating (2) and cell-entry (3) properties, or the classification of T-cell epitopes (4). However, the mode of action of a peptide sequence depends on a variety of biochemical factors, which cannot be reflected by the order of the AAs alone (1). Moreover, many machine learning models require a numerical input with a fixed dimension (5). To this end, many descriptors, i.e. sequence-based encodings (SeBEs) have been developed, aiming to compute adequate numerical representations of the primary structure. In short, SeBEs are algorithms mapping the AAs to numerical values, but also incorporate interactions of non-adjacent residues, for instance, by autocorrelation techniques (6,7). SeBEs have been successfully employed in numerous studies, for example, for the applications mentioned above, but also to predict antiviral (8) or anticancer peptides (9). In addition, tools such as *iFeature* (6) or *BioSeq-Analysis2.0* (10), which allow easy access to SeBEs, have paved the way for a wide range of biomedical applications.

However, the function of a peptide is not only defined by its primary structure, but biological meaning will be also encoded in higher dimensions, i.e. the peptide’s secondary or tertiary structure. Consequently, structure-based encodings (StBEs) augment SeBEs to maximize the information gain. StBEs can be divided into two further groups: encodings derived from the secondary structure and those derived from the tertiary structure. The former includes encodings describing, for example, the α-helix composition (6), based on an *ab initio* secondary structure prediction (11). For the latter, Bose *et al.* (2011) utilized the Delaunay triangulation to encode protein structures as numerical feature vectors (12). The aim of the study was to predict protein structure properties and the results showed, that this StBE is capable to preserve tertiary structure information for machine learning purposes (12). Furthermore, Löchel *et al.* (2018) demonstrated, that using the electrostatic hull of V3-loop of the gp120 protein, substantially improved the prediction of co-receptor tropism of the human immunodeficiency virus 1 (13). A comprehensive introduction to encodings, specifically dealing with the prediction of antimicrobial peptides, can be found in our recent review (7).

Nevertheless, several major challenges remain. First of all, there is no guideline or clear recommendation which encodings work well for specific biomedical applications, facing researchers with the effort of matching the right encoding for the task. Second, even if one or more encodings have been determined, researchers are very likely challenged with parameterized ones, further increasing the hyperparameter search space and thus, actually aggravating the encoding exploration. Third, many studies confirmed that combining different encodings to ensemble classifiers, effects the prediction performance positively (14,15). Specifically, Dybowski *et al.* (2011) employed stacked generalization on the predictions of SeBE- and StBE-based classifiers and thus, improved the resistance prediction to Bevirimat, an antiretroviral drug class (16). Consequently, applying ensemble learning techniques enlarges the hyperparameter search space further and a structured exploration becomes more and more difficult.

For this reason, we present here, to the best of our knowledge, the first large-scale comprehensive study on state of the art peptide encodings on a wide range of datasets from a wide range of biomedical domains. Our study closes the gap between the availability of a great variety of encodings and the important question whether one of them is best suited for a specific domain application or task. This study builds upon our recent review on peptide encodings (7), which allows us to add additional, literature-known sequence- and structure-based encodings. The goal of the study is to provide researchers, faced with a biomedical classification task at hand, general guidelines, which encodings are likely to be superior on a certain biomedical classification task. Thus, we investigated the two major encoding types, namely SeBEs and StEBs, in total leading to 48 encoding groups. Moreover, we collected 50 datasets from multiple domains, including antimicrobial, -viral and -cancer as well as cell-penetrating peptides as already mentioned above, but also from further fields, such as HIV drug resistance prediction. By further taking the parameterization of some of the encoding groups into account, we generated altogether hundreds of thousands of encoded datasets.

**Figure 1.**
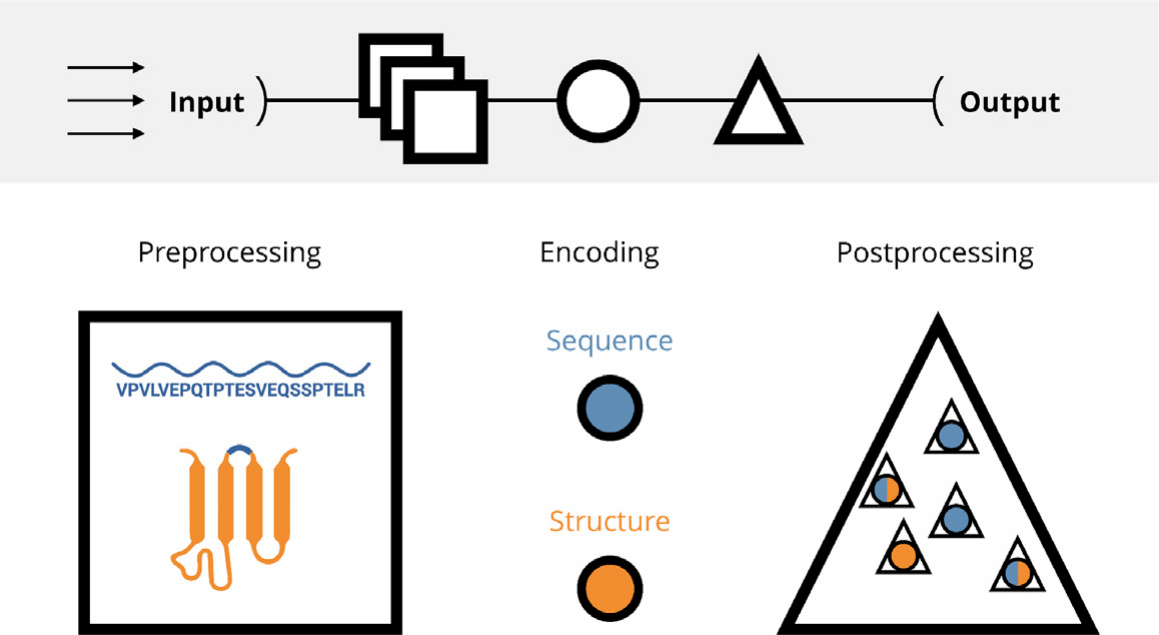
The general principle of the PEPTIDE REACToR. The emphasis is put on a high-throughput processing of an arbitrary amount of input datasets (arrows), followed by the preprocessing, encoding, and postprocessing, generating the final output (top). The preprocessing includes sanitizing of the input sequences, the filtering and the tertiary structure approximation (squares). Afterwards, the sequences as well as the accompanied structures are used for the encoding (circles). The postprocessing involves the machine learning and the actual benchmarking including the visual preparation of the analyses (triangles).

To meet this unique challenge we have developed the PEPTIDE REACToR, a platform bundling manifold analyses to examine characteristics of the encoded datasets (see Figure 1). The workflow is designed for high parallelization, enabling an efficient evaluation, even in the case of additional encodings and datasets in the future. Surprisingly, our results point out, that no particular encoding can be recommended in general. However, there are encodings that show increased performance across multiple datasets, hence, biomedical domains. Contrary, our method reveals many inferior encoding groups, questioning the necessity of computing them at all. Thus, our findings pave the way for automated machine learning approaches, in that the hyperparameter space is drastically reduced and relevant techniques become computationally feasible. According to the FAIR data principles (findability, accessibility, interoperability and reusability) (17), the results can be interactively accessed at https://peptidereactor.mathematik.uni-marburg.de/ and all datasets can be downloaded at a central location. The source code as well as the datasets are available at https://github.com/spaenigs/peptidereactor.

## MATERIALS AND METHODS

We collected 50 datasets from a wide range of biomedical applications. Furthermore, building upon our recent encoding review (7), we aggregated in total 48 encodings and developed a high-throughput approach facilitating a parallelized encoding and the subsequent comparison of the encoded datasets. Every task is part of a large-scale, end-to-end workflow and will be executed automatically. An overview of the workflow can be found in Figure 1. We used Python v3.7.4 (https://www.python.org/) and R v3.5.2 (https://www.r-project.org/) throughout the analysis. The pipeline itself as well as the algorithms in particular have been implemented as a modular Snakemake v5.19.0 (18) pipeline. Moreover, we used Scikit-learn v0.23.1 for the machine learning algorithms and validation metrics (19).

The following sections describe the applied methodology by keeping the actual order of the workflow. Thus, the dataset collection will be presented at first. The subsequent section introduces the tertiary structure approximation, since it is crucial before the actual encodings and their properties are presented. Some of the encodings are parameterized, thus, leading to thousands of encoded datasets. Therefore, the next section sheds light on the algorithmic details of the encoded datasets filtering. Finally, the actual benchmark methodology will be presented and the method section concludes with the result visualization description. Refer to Figure 1 for a visual summary.

### Datasets collection

We collected 50 different datasets comprising peptides and small proteins from various biomedical domains. These include immunomodulatory and cell-penetrating peptides, but also peptides specifically targeting cancer, fungi, microbes, tuberculosis and viruses. Moreover, we added datasets from HIV research specifically covering resistance prediction against different drug classes and protease cleavage site prediction. A further application refers to the detection of neuropeptides as well as a- and b-cell epitopes. More attributes, for example, origin, size, etc. can be found in the Supplementary Table S1. Detailed dataset descriptions are specified in Supplementary Table S3.

The datasets were composed for manifold reasons. They reflect a broad field of action, including infectious diseases, for example, HIV, antimicrobial resistance of multi-drug resistant bacteria, and others, to elevate the significance of the results. If possible, we used several datasets per domain to also reflect the sequence diversity. In order to cope with the high-dimensionality of the present study, we limited the benchmark to two-class problems.

**Figure 2.**
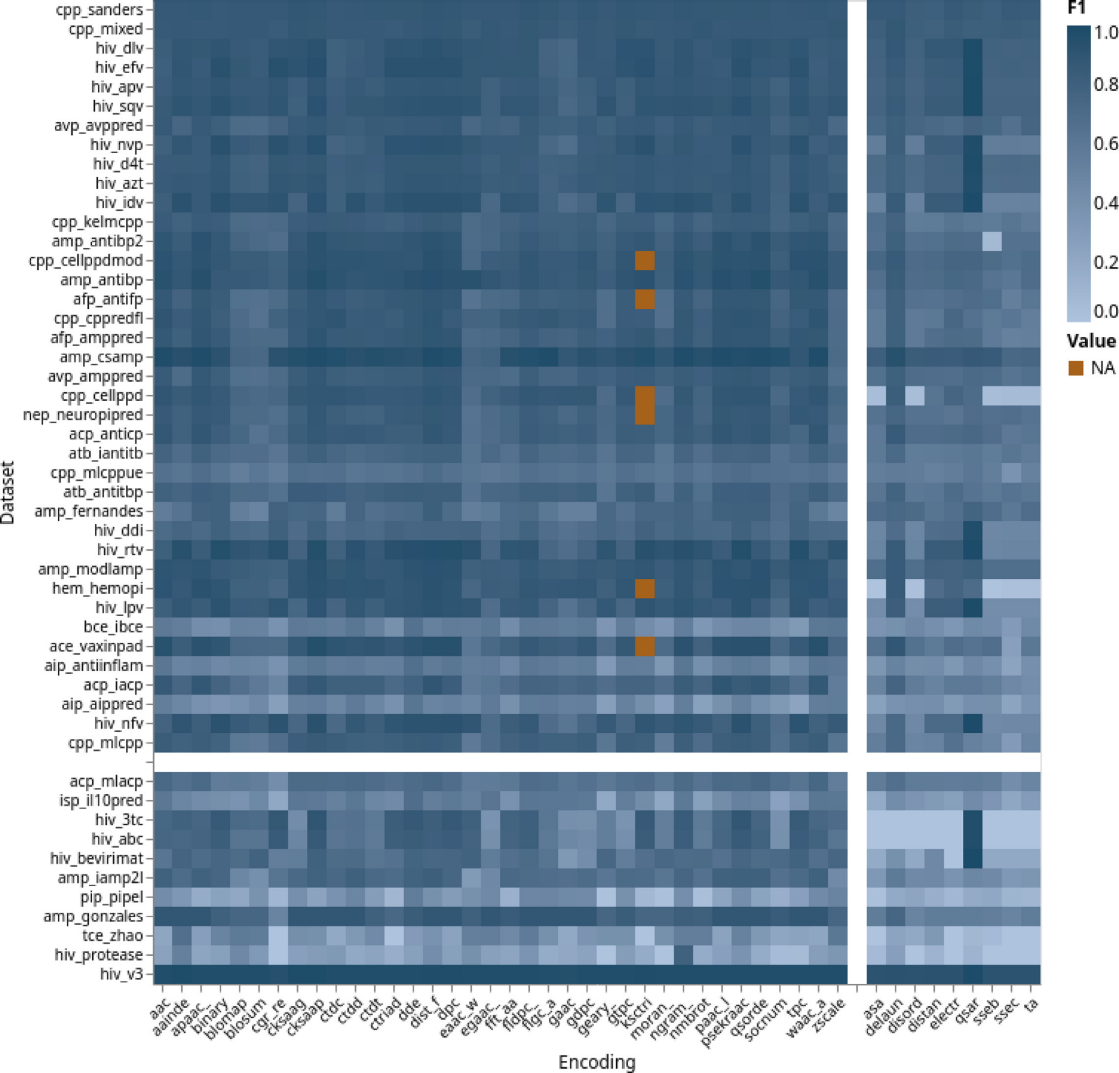
Encoding groups performance, sorted by class imbalance and encoding type. Color coding corresponds to the maximum F1-score of the bootstrapped medians for a group. The *x*-axis is organized by sequence- and structure-based encodings. The *y*-axis is sorted by class imbalance (cut-off 0.35). Groups are separated by white bars. An interactive version of this plot can be found at https://peptidereactor.mathematik.uni-marburg.de/.

Moreover, the datasets have been applied largely as they are, in order to stay as close as possible to the original usage. That is, the class ratio of the datasets at hand ranges from well balanced (e.g. *ace_vaxinpad*) to very imbalanced ones (*hiv_v3*) (see Figure 2). This affects also the size of the datasets, which ranges from small ones (e.g. *amp_gonzales*) to relatively large datasets (e.g. *amp_iamp2l*). Refer to Supplementary Tables S1 and S3 for more details. Too large datasets have been excluded from the study or, if present, the validation dataset were used instead.

All in all, the datasets are composed of 53 041 sequences ranging from 3 to 255 amino acids. The mean sequence length is 55.04 (±67.58) with a median length of 26 amino acids. Refer to Supplementary Table S2 for a comprehensive descriptive evaluation on the datasets used in this study. In particular, let *D*_*i*_ be the *i*-th dataset from a biomedical application, i.e. composed of a set of *n* amino acid sequences *s* of length *k*, denoted as

(1) $$\begin{equation*} D_i=\lbrace s_1,s_2,\dots ,s_{n-1},s_n\rbrace \end{equation*}$$

and

(2) $$\begin{equation*} s_i=\lbrace a_1,a_2,\dots ,a_{k-1}, a_k\rbrace \end{equation*}$$

with *a*_*i*_ being one of the 20 natural amino acids.

### Tertiary structure approximation

Two categories of encodings have been investigated: sequence- and structure-based encodings (SeBEs and StBEs, respectively). While the former are derived from the primary structure, i.e. the amino acid sequence, the computation of the latter bears on the secondary, if not the tertiary structure of a peptide or protein, respectively. Even though algorithms exist for the prediction of secondary structure properties, for example, SPINE X (20), or the prediction of the tertiary structure, for instance, RaptorX (21), they are often computationally expensive, above all, if one aims to predict hundreds of structures simultaneously.

However, for a large-scale approach, this is not practical, thus, we developed in addition an algorithm, which approximates the tertiary structure for later usage by StBEs. While PSI-BLAST (22) is capable of finding more distant relative sequences, it often suffers from a long run time for long sequences. Thus, we applied BLAST (23) v2.9.0 instead. In order to set up a database, we downloaded all available structures (as of May 2020) from the Protein Data Bank (24) (PDB, http://www.rcsb.org/), extracted all sequences into a FASTA file using Biopython v1.7.4 (25,26) and used it as input for the *makeblastdb* command. By doing so, we ensure, that the database contains only sequences with a known structure.

For a sequence *s*_*i*_, the structure approximation works as follows: first, an initial BLAST run tries to find the query sequence within a PDB entry. For the best match, i.e. the match with the lowest e-value, the respective PDB file will be fetched. The algorithm clips the matching part from the structure and returns the *i*-th tertiary structure approximation for a query sequence *s*_*i*_. Any *s*_*i*_, for which no structure has been found, is omitted in the later encoding step.

### Encodings

Spänig and Heider (2019) conducted an extensive literature search and collected a wide range of SeBEs and StBEs (7). We employed the Python package *iFeature*, which already provides many encodings (6). Moreover, we also added the frequency matrix chaos game representation (FCGR), an adoption of the original CGR, recently developed by our group (27). However, as part of this study, we contribute the implementation of 10 additional encodings to the scientific community, i.e. encodings, which have been used successfully in the literature, but where an actual implementation is lacking. For a comprehensive list of all encodings, refer to Supplementary Table S4. Supplementary Note S1 provides the algorithmic details on the additional encodings, for the remainders, refer to (6) or (7). In addition, we employed MUSCLE v3.8.1551 (28) in case an encoding, for instance, the binary encoding, requires a multiple sequence alignment beforehand. In particular, an encoding is a function *f*, mapping an amino acid sequence *s*_*i*_ to an numerical vector $\hat{s}_i$:

(3) $$\begin{equation*} f:s_i\rightarrow \hat{s}_i, \hat{s}_i \in \mathbb {Q}^\mathbb {N} \end{equation*}$$

### Filtering

Since some of the encodings are parameterized, thus, leading in total to thousands of encoded datasets, an important part of the pipeline is the filtering of the *d* encoded datasets $\lbrace \hat{D}_1,\dots ,\hat{D}_d\rbrace$, hence to reduce the extent of *d* before the actual benchmark. For the purpose of a benchmark, we covered the input parameter space for all encodings as extensive as possible, thus we generated in total *d* encoded datasets:

(4) $$\begin{equation*} d = \sum _{i}^{48}|\overrightarrow{x_1(i)}\times\cdots \times\overrightarrow{x_n(i)}| \end{equation*}$$

Whereby × denotes the Cartesian product and *n* refers to the *n*-th parameter set for the *i*-th encoding group. Specifically, the amino acid index-based encodings are highly related owing to an intrinsic correlation of certain amino acid indices. Moreover, parameterized encodings take the window length $\vec{\boldsymbol {w}}$ of size *k* for autocorrelation-based encodings, or correlation types $\vec{\boldsymbol {c}}$ of size *l*, tuple sizes $\vec{\boldsymbol {t}}$ of size *m*, and gap length parameters $\vec{\boldsymbol {g}}$ of size *n* for the pseudo K-tuple reduced amino acids composition (PseKRAAC) encoding leading to $\vert \vec{\boldsymbol {w}} \vert + \vert \vec{\boldsymbol {c}} \times \vec{\boldsymbol {t}} \times \vec{\boldsymbol {g}} \vert$ encoded datasets for these encodings groups alone. Refer to Supplementary Table S4 for the comprehensive list on parameterized encodings and which parameter space have been covered in particular. Supplementary Note S4 provides a detailed description of the filter algorithm for the amino acid index as well as PseKRAAC encodings.

### Benchmark

The essential part of this project is the high-throughput evaluation of all encodings across multiple biomedical datasets. To this end, several advanced processing as well as analysis steps are conducted, which are introduced more detailed hereinafter.

#### Model training

In order to standardize the analysis, we used the Random Forest classifier (RFC) (29) with default parameter settings as the default machine learning model. RFCs already perform good without hyper-parameter optimization, which is contrary to, for example, Support Vector Machines, which achieve far greater performance with optimized hyper-parameters compared to the defaults (30). That is, RFCs are more stable, allowing us to neglect the influence of hyper-parameter optimization on the encoding performance. Moreover, we chose this classifier since it exhibits a variety of advantages compared to other prediction models. It internally picks the most predictive features out of a set of multiple decision trees, that is, it has a built-in feature selection method. Moreover, the final prediction is based on the trees built from the selected features; hence, it is also an ensemble algorithm. In addition, the feature importance can be calculated and the RFC is also capable of reducing overfitting (29).

#### Cross-validation

In order to generalize the model performance, we applied a repeated, stratified *k*-fold cross-validation (CV). In particular, for each validation round, an encoded dataset $\hat{D}_{i}$ is splitted into *k* = 5 folds and each CV is repeated 10 times. For each fold, the intermediate results, i.e. the vectors of the predicted classes $\vec{\boldsymbol {t}}$, the probabilities $\vec{\boldsymbol {p}}$ and the actual classes $\vec{\boldsymbol {y}}$ are stored in the matrices $\boldsymbol {R_p}$ and $\boldsymbol {R_t}$, whereby $p\in \lbrace \vec{\boldsymbol {t}},\vec{\boldsymbol {p}}\rbrace$ and $t\in \lbrace \vec{\boldsymbol {y}}\rbrace$. $\boldsymbol {R_p}$, analogous to $\boldsymbol {R_t}$, is denoted as shown in Equation 5, with $p_{fold_k,pred_n}$ being the *n*-th predicted probability or class of the *k*-th fold. In addition, the number of rows in both matrices corresponds to the repetitions as well as folds of the CV, hence 50 in the present case.

(5) $$\begin{equation*} \boldsymbol {R_p}=\left[ {\begin{array}{*{10}c}p_{fold_1,pred_1} & \dots & p_{fold_1,pred_n}\\ \vdots & \ddots & \vdots \\ p_{fold_k,pred_1} & \dots & p_{fold_k,pred_n}\\ \end{array}} \right] \end{equation*}$$

Note, that the overall CV is conducted two times: one time for each $\hat{D}_{i}$ without any restrictions and a second time for two groups of encodings, for example, for SeBEs and StBEs. As mentioned above, it might be the case, that a tertiary structure approximation failed. Consequently, a dataset $\hat{D}_i$, based on a StBE, might lack certain sequences, but the two-group CV needs to ensure equal records in both $\hat{D}_{i}\in$ SeBEs and $\hat{D}_{k}\in$ StBEs. Thus, in the case of a two-group CV, we compute the intersection of the record labels and remove the additional rows from $\hat{D}_{i}$ prior to the actual CV.

#### Performance metrics

In order to evaluate the performance of the encodings with a single measure, we calculated the following metrics: F1-score, Matthews Correlation Coefficient (MCC), Precision, Recall (Sensitivity) and Specificity. Each of these measures has particular properties, allowing them to highlight the advantages or disadvantages of specific encodings concerning the task. Refer to Supplementary Note S2 for the respective formulas. All metrics are computed on the *k*-th split of the *k*-th row from $\boldsymbol {R_p}$ and $\boldsymbol {R_t}$.

#### Similarity

The similarity of classifiers, that is, the similarity of the predictions of unknown test examples from the respective classifiers, trained with the encoded datasets $\hat{D}_i$ and $\hat{D}_j$, could stress specific strengths and weaknesses of an encoding. To this end, we implemented two similarity measurements, namely the Phi coefficient (31) (see Supplementary Note S2) and the disagreement measure *D* (31,32), with the respective output of the *i*-th classifier *o*^*i*^_*k*_ and of the *j*-th classifier *o*^*j*^_*k*_, denoted as:

(6) $$\begin{equation*} D_{i,j}=\frac{1}{n}\sum _{k=1}^{n} \left| o{^i}_k-o{^j}_k \right| \end{equation*}$$

Analogous to the performance metrics, we computed the particular similarity for the *k*-th CV split on the *k*-th row of the *i*-th and the *j*-th classifier outputs $\boldsymbol {R{^i}_p}$ and $\boldsymbol {R{^j}_p}$, respectively. Finally, the overall similarity is the average across all splits. The two-group CV is the basis for the similarity measures since the output of the classifiers *i* and *j*, need to be traceable to the same sequences.

#### Critical difference

There are several statistical tests for evaluating machine learning models trained on multiple datasets. Depending on the classification task at hand, Santafé *et al.* (2015) provided an overview of the recommended procedure (33). In the present case, we considered the models trained on many encoded datasets as the statistical comparison of several classifiers trained on several datasets. In particular, we assume, that using the RFC models trained on *k* encoded datasets instead of *k* algorithms fulfills the criteria for the Friedman statistic χ^2^_*F*_, meaning the models are related, i.e. paired, and each fold is independent of each other:

(7) $$\begin{equation*} \chi {^2}_F=\frac{12N}{k(k+1)}\left[ \sum _{j} R{^2}_j-\frac{k(k+1)^2}{4} \right] \end{equation*}$$

with the the Iman and Davenport correction:

(8) $$\begin{equation*} F_F=\frac{(N-1)\chi {^2}_F}{N(k-1)-\chi {^2}_F} \end{equation*}$$

in order to verify, whether one of the models outperforms another. That is, to reject the null hypothesis, which states, that there is no difference between the classifiers. In particular, the Friedman test compares the ranks *r*^*j*^_*i*_ of the *j*-th model validated on the *i*-th fold. The average rank is denoted as $R_j=\frac{1}{N}\sum _{i}r{^j}_i$ calculated on *N* folds and *k* trained classifiers using *k* − 1 degrees of freedom. Moreover, *F*_*F*_ is F-distributed with *k* − 1 and (*k* − 1) (*N* − 1) degrees of freedom (34).

The alternative hypothesis states, that there is a statistically significant difference across the models. In the case of acceptance, the post-hoc analysis using the Nemenyi test unveils the significantly different models. Hence, the critical difference *CD*, denoted as

(9) $$\begin{equation*} CD=q_\alpha \sqrt{\frac{k(k+1)}{(6N)}} \end{equation*}$$

is computed using the critical value *q*_α_, which is based on the Studentized range statistic with *k*(*N* − 1) degrees of freedom and a significance level of α = 0.05. Two classifiers perform significantly different, if $|R_j-R_{\hat{j}}|\ge CD$ (34).

The statistical tests are implemented as part of the R-package *scmamp* v0.2.55 (35).

#### Encoding correlation

As already pointed out in a previous section, many encoded datasets are either intrinsically correlated, for instance, the AAI-based encodings or derived from the same encoding group, but with slightly different parameters, for example, the window size. Ultimately, we are dealing with high-dimensional, potentially very similar datasets of varying dimensions. In order to measure the degree of correlation, we utilized the adjusted RV-coefficient, which has been developed for these particular case (36):

(10) $$\begin{eqnarray*} RV_{adj}(\boldsymbol {X},\boldsymbol {Y})=\frac{\sum _{i=1}^{p}\sum _{j=1}^{q}r{^2}_{adj}(x_i, y_j)}{\sqrt{\sum _{i,j=1}^{p}r{^2}_{adj}(x_i, x_j)\sum _{i,j=1}^{q}r{^2}_{adj}(y_i, y_j)}} \nonumber\\ \end{eqnarray*}$$

with *r*^2^_*adj*_(*x*, *y*) being the adjusted Pearson correlation coefficient (see Supplementary Note S4, Equation S10) between two feature vectors, denoted as:

(11) $$\begin{equation*} r{^2}_{adj}(x,y)=1-\frac{n-1}{n-2}(1-r^2(x,y)) \end{equation*}$$

Moreover, $\boldsymbol {X}$ and $\boldsymbol {Y}$ refer to the encoded datasets $\hat{D}_i$ with *p* and $\hat{D}_j$ with *q* features as well as *n* encoded sequences, respectively. The *i*-th feature vector from $\boldsymbol {X}$ is denoted as *x*_*i*_ and the *j*-th feature vector from $\boldsymbol {Y}$ is denoted as *y*_*j*_. Indahl *et al.* (2015) implemented the adjusted RV-coefficient as part of the *MatrixCorrelation* R-package, which we utilized in version 0.9.4 (37). Since an all versus all calculation is computationally expensive, we determined the *RV*_*adj*_ only for the top 50 encodings, based on the F1-score.

### Encodings across multiple domains

For the comparison of encodings across multiple datasets, i.e. biomedical domains, we merged the encodings into groups (see Supplementary Table S4) and considered the best-performing encoding (average F1-score of the CV results) as the group representative. Based on these, we ranked the encoding groups across all datasets in order to uncover domain-specific patterns. Moreover, we clustered the datasets and encoding groups by means of the hierarchical clustering using the UPGMA (Unweighted Pair Group Method with Arithmetic mean) method with the euclidean distance (38). We used the implementation provided by the SciPy package (39).

Moreover, for each dataset $\hat{D}_i$, encoded via the amino acid composition encoding, we applied t-SNE with default settings on the sequences of the positive class as well as for both classes. Thus, each $s_i^+$ and *s*_*i*_ is embedded in the same two-dimensional space, allowing insights specifically regarding the sequence similarity within various biomedical domains and the diversity of the datasets on the sequence level.

### Data visualizations

The results are visually depicted and summarized by means of *Altair* v4.1.0 statistical visualization library (40). In particular, we plotted the results for analyzing two kinds of categories (single datasets and summary graphics for all encoded datasets). We followed the 10 simple rules on how to colorize biological data visualizations and applied them in our workflow (41). Note, that in general the choice of the top encodings is made due to the corresponding F1-score. Refer to the Supplementary Note S3 for more details. Finally, the visualizations are aggregated into an interactive report, which can be found at https://peptidereactor.mathematik.uni-marburg.de/.

## RESULTS

### Workflow

The PEPTIDE REACToR features high-throughput capabilities and a modular design, allowing the processing of an arbitrary amount of encodings and datasets. Novel encodings and additional datasets can be investigated, making it sustainable and future-ready. The benchmark is set up as a high-throughput, large-scale Snakemake (18) workflow. In particular, it is implemented with three important goals in mind: first, efficient use of the available computing power, second, a high parallelization and third, make it findable (F), accessible (A), interchangeable (I) and reusable (R), according to the FAIR data principles (17). However, as the different preprocessing, encoding, as well as benchmark tasks are very diverse and the implementation as one large workflow is cumbersome, the workflow has been designed in a way, that multiple meta nodes, responsible for a specific task or algorithm, are aggregated to a meta workflow. Each meta node is a Snakemake pipeline itself, exposing a defined application programming interface (API), thus, making them interchangeable and reusable. For an easy setup and high reusability, the meta workflow is executed within a Docker v19.03.2 (https://www.docker.com/) environment using Conda v4.8.3 (https://docs.conda.io/en/latest/) for package management.

### Performance

In general, the performance of the SeBE groups are superior to the StBE groups (see Figure 2). As an exception, the *qsar* encoding works better on some of the *hiv* datasets. We also observed an increased performance on datasets with relatively balanced class sizes, i.e. the more imbalanced a dataset, the poorer the performance. The *hiv_v3* dataset is an exception. Albeit its striking imbalance, i.e. 200 versus over 1000 sequences for positive and negative class, respectively, the performance of all encodings is good. In addition, we were not able to observe specific encoding groups that are more powerful on certain biomedical classification tasks (see Supplementary Figure S1). The performance does not seem to follow a specific pattern. For instance, all encoding groups showed average performance on the *cpp_mlcppue* dataset, although the classification of the remaining *cpp* datasets was clearly better.

#### Ranks

**Figure 3.**
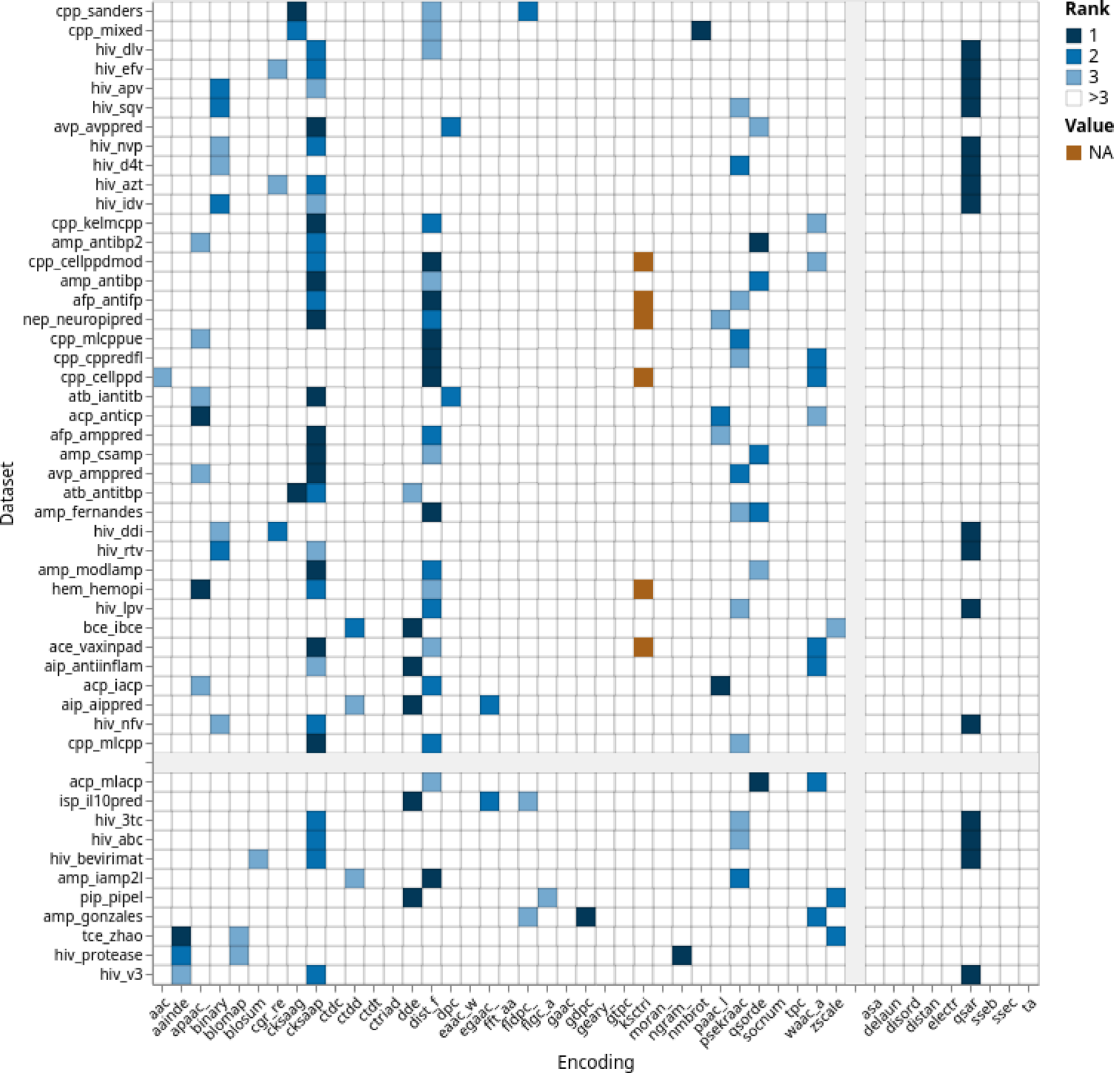
Ranked encoding groups performance, sorted by class imbalance and encoding type. Color coding corresponds to the ranks of encodings across datasets. The x-axis is organized by sequence- and structure-based encodings and the y-axis is sorted by class imbalance (cut-off 0.35). Groups are separated by gray bars. An interactive version of this plot can be found at https://peptidereactor.mathematik.uni-marburg.de/.

Three groups stood out: the *cksaap*, the *distance_frequency* and the *qsar*-based encodings (see Figure 3). Encodings within these groups were more often among the top 3, compared to encodings from the remaining ones. In contrast, the majority of the encoding groups, in particular StBE groups, were rarely among the best.

#### Clustering

**Figure 4.**
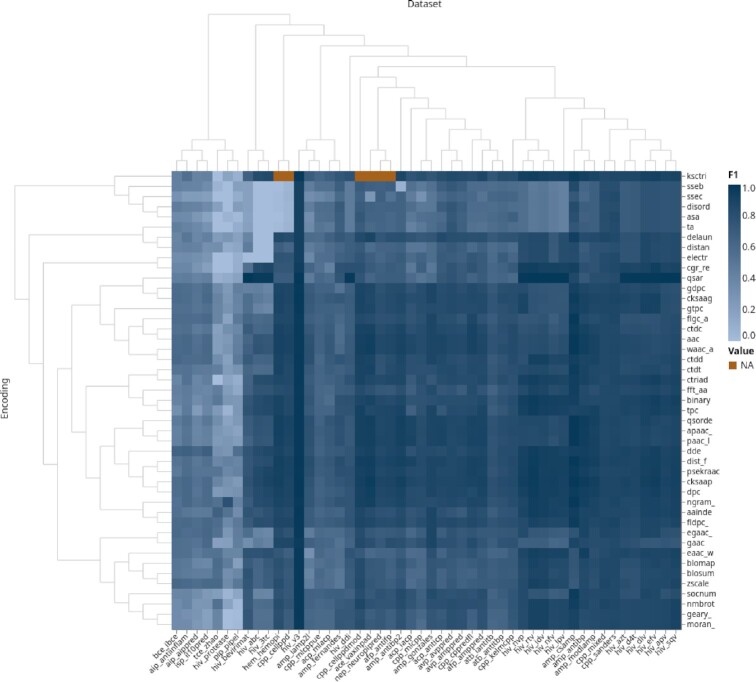
Encoding groups performance, clustered by biomedical domain and encoding group. Color coding corresponds to the max F1-score of a group. The *x*-axis is arranged by clustering datasets, i.e. the biomedical application. The *y*-axis is organized by clustering sequence- and structure-based encodings. An interactive version of this plot can be found at https://peptidereactor.mathematik.uni-marburg.de/.

An automated clustering confirmed our findings mentioned above. One can observe two major clusters for the encoding groups and datasets, respectively (see Figure 4). The encoding ones include mainly the SeBE and StBE groups. The former can be further distinguished in three sub-clusters, ranging from (i) the *qsar* to the *ctdd*, (ii) the *ctdt* to the *fldpc_*, as well as (iii) the *egaac_* to the *moran_* encoding groups, although no real pattern emerges within these. An exception are encodings based on the dipeptide composition, namely the *dde*, *dpc* and the *fldpc_* encoding, as these are all within the second cluster. However, the *gdpc* encoding can be found in the first cluster.

Regarding the dataset clusters, the larger of the two can be divided again into three parts, namely (i) from the *hiv_bevirimat* to the *hiv_ddi*, (ii) the *cpp_cellppdmod* to the *atb_antitbp*, and finally (iii) from the *cpp_kelmcpp* to the *hiv_sqv* datasets. Albeit the latter includes predominantly *hiv* related datasets, in general no actual patterns can be observed within the groups. In addition, a two-dimensional embedding of the sequences of the positive class explains some of the dataset clusters (see Supplementary Figure S2). One example is the grouping of the *hiv_nfv*, *hiv_rtv* and *hiv_idv* datasets. The sequences of these datasets form similar, compact clusters.

#### Median performance

A closer examination of the encodings reveals groups where the range spanned between the worst and the best encoding is noticeable, meaning the best encodings show similar performance compared to the top encodings across all groups and vice versa (see Supplementary Figure S4). In addition, the StBEs show in general worse performance compared to the SeBEs. This can be verified by considering the metrics in detail (see Supplementary Figures S5 and S6). StBEs are mainly located more to the right, i.e. showing a smaller value of the respective metric. However, by comparing adjacent encodings in Supplementary Figures S5 and S6, we found no significant differences (42). Furthermore, some of the outliers explain the gap between the best and the worst encodings, mentioned above. Overall, encodings from the same group are frequently among the best encodings, i.e. if two encodings are derived from the same group, but with different parameters, the performance is similar. By considering the receiver-operation characteristic (ROC)- and the Precision-Recall (PR)-curve areas of the overall top 6 encodings as well as the top 3 SeBEs and the top 3 StBEs, the observations mentioned above can be further endorsed (see Supplementary Figure S7).

### Similarity

The similarity of the classifier outputs based on the Phi correlation indicates that encodings within groups and similar performing ones reveal a higher correlation (see Supplementary Figure S8). This can be verified by specifically considering SeBEs versus StBEs, which show in general a lower similarity. Furthermore, the diversity of the predictions, i.e. the disagreement measure of the classifier outputs, underpins these observations, since similar encodings as well as similar outputs leading to a lower diversity, hence greater similarity (see Supplementary Figure S8).

#### Class separation

With this respect, considering not only the diversity but also the probabilities predicted by a particular encoding combination, one can observe that the clustering quality, i.e. the classification capability of two encodings, measured by the Davis-Bouldin score (DBS), is often dependent on the diversity. In particular, by combining a well-performing SeBE and StBE, which show higher diversity compared to the best group-independent encodings, an increased DBS, hence better class separation, can be observed for the former (see Supplementary Figure S9).

However, this is not always the case (see Supplementary Figure S10). Albeit the encoding diversity and the DBS of the clusters are related, the DBS seems to increase only until a particular diversity, meaning, that a too diverse classifier output negatively affects the class separation furthermore.

#### Critical difference

The observations made by the similarity measurements are further statistically revised by the critical difference of the respective classifier outputs. The critical difference unveils a great variety of encodings, which are not significantly different (see Supplementary Figure S11). Like above, this can be specifically observed for encodings from one group, which is in accordance with the previous experiments. However, the *psekraac_* and the *ngram_* groups are an exception (see Supplementary Figure S11). In addition, encodings, which surpass the critical threshold by several orders of magnitude, are less present (see Supplementary Figure S11).

#### Dataset correlation

**Figure 5.**
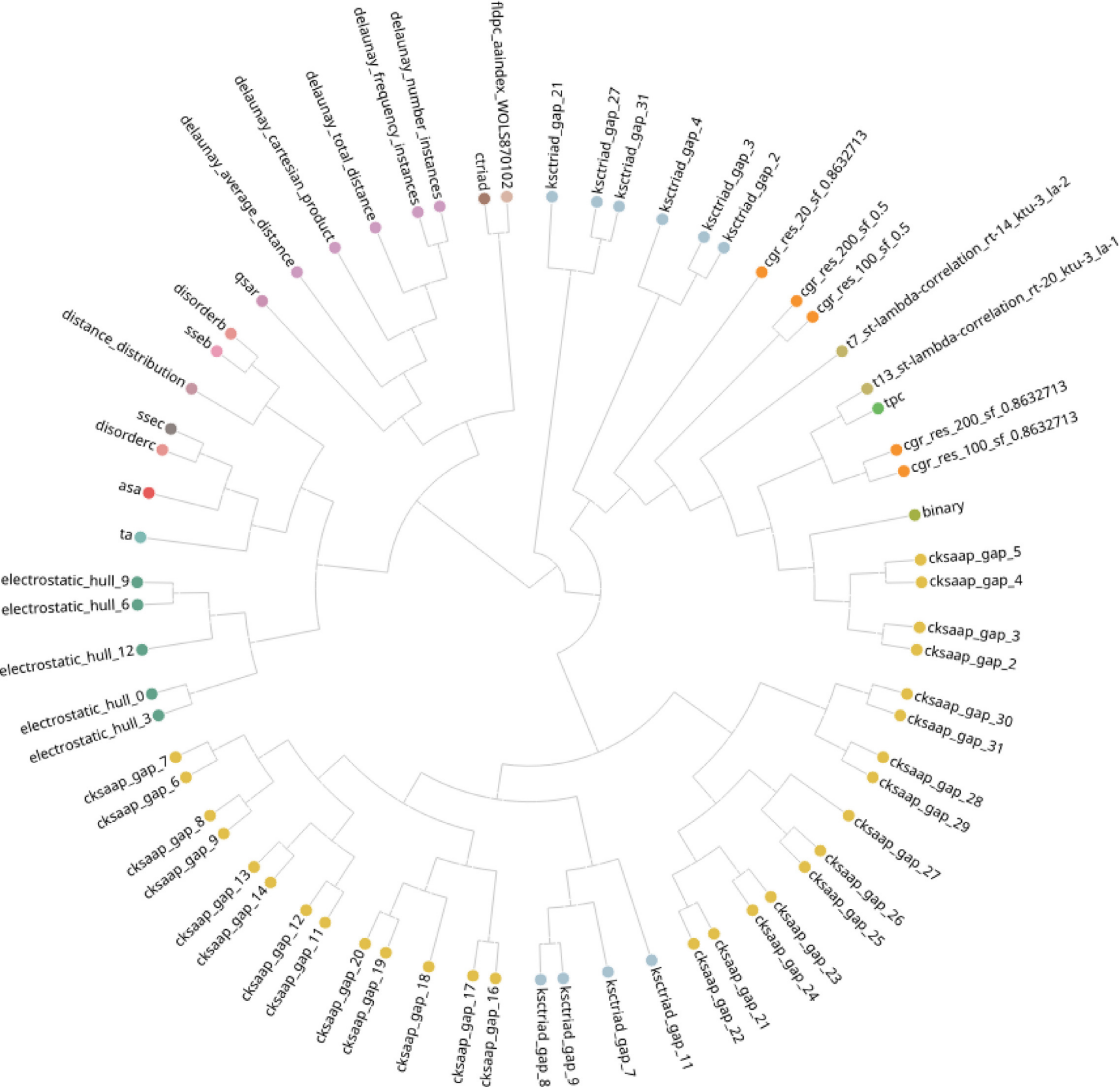
Top 50 datasets correlation. Correlation of top 50 encoded datasets based on the adjusted RV-coefficient. Color coding corresponds to the encoding group. The graphic shows the example of the *hiv_ddi* dataset. The online version of this plot can be found at https://peptidereactor.mathematik.uni-marburg.de/.

Finally, the measured correlations, solely based on the encoded datasets, verify our observations made throughout the analyses (see Figure 5). The results illustrate foremost that encodings, originating from the same group, are clustered in separate branches. In addition, considering specifically StBEs, also here a clustering in an own sub-branch can be observed. This is in agreement with our findings from above, i.e. similar encodings are jointly clustered and thus, their predictions are also often related significantly.

### Encoding recommendation

Based on our results elaborated above, we are not able to determine encodings, which can be specifically recommended for a particular application. However, following our findings a general guideline can be provided:

1. Some of the encoding groups are often among the top 3. Refer to Figure 3 for an overview and to which this applies in particular. Encodings from these groups are in general superior and should be preferably applied.
2. SeBEs are faster to compute and show in general a higher performance; thus, they should be preferred over the StBEs (see Figure 2 and Supplementary S13). However, combining SeBEs and StBEs to an ensemble classifier could outperform single SeBEs (see (7) and Supplementary Figure S9).
3. The dataset size should be also considered (see Supplementary Figure S12), i.e. we recommend for larger ones to carefully deliberate the choice of encodings. Contrary, for smaller datasets all encodings can be computed without hesitation.
4. A few encodings show better performance on imbalanced datasets. Refer to the Figure 3 for an overview and to which encodings/datasets combination this applies to.
5. Consider the size parameter for autocorrelation-based encodings (*cksaagp*, *cksaap*, *socnumber*, *qsorder*, *nmbroto*, *moran_*, *ksctriad*, *geary_*, *eaac*, *apaac_*, *paac*, *egaac_*, and *psekraac*). Shorter sequences require a smaller, for example, window size and vice versa.
6. Select solely one particular encoding from a parameterized encoding group. Encodings from the same group often show a similar performance (see Supplementary Figures S5, S8, and S11). This is due to highly correlated encoded datasets (see Supplementary Figure S5).
7. Use ensemble methods and aggregate different encodings to a meta learner in order to improve the performance.
8. For encodings that are seemingly relevant for a specific task, but fail in practice, extend the encoding choice iteratively, i.e. be less stringent with respect to the points mentioned above, in order to find encodings with improved performance.

## DISCUSSION

We presented here, to the best of our knowledge, the first large-scale comprehensive study on peptide encodings. In particular, we aggregated numerous sequence- and structure-based encodings (SeBEs and StBEs, respectively) as well as datasets from a wide range of biomedical domains. Albeit proteins and peptides may exhibit multi-functionality (43), we limited our case study to two-class classification tasks. Hence, we can exclude that an insufficient size of the respective classes affects the prediction negatively, ultimately decreasing the complexity of this work and allowing for more robust conclusions.

The choice of the Random Forest classifier (RFC) as the default machine learning model also reduces the complexity. A hyper-parameter optimization (HPO) is less important as it would be for other models (30). In addition, the built-in feature selection discards irrelevant features, thus RFCs standardize the pre-condition for all encodings. This also reflects applied machine learning, where feature selection is a standard measure and encodings would be ultimately assessed based on their representative feature subset. Nevertheless, HPO (including the choice of the classifier) has the possibility to impact the encoding performance slightly. In order to cope with the computational feasibility we omitted an in-depth HPO. However, further research is necessary to address the impact of HPO on the encoding performance.

All in all, our study closes the gap between a broad range of peptide encodings and the challenge which to use on a specific biomedical dataset. We observed that no particular encoding group shows superior performance within a biomedical domain, i.e. no general pattern emerged from the respective encoding performance. However, insights are hereinafter discussed in more detail.

### Performance

The encoding performance depends on two main characteristics. First, the class imbalance and second, the type, i.e. SeBE or StBE. While the former is not surprising, as it needs more sophisticated measures for coping, the second is potentially due to the initial tertiary structure approximation. Thus, in many cases, the structure is probably unrelated with the *in vivo* one. In contrast, for the database, we used only sequences with a known structure deposited at the PDB. This could be the reason, why the general performance of StBEs is lower compared to SeBEs, but the predictions are still satisfying. We suspect that disordered regions also affect the prediction negatively, since no conformational information can be derived from it.

A further reason for a convincing prediction is the sequence similarity within and across the positive and the negative class. Regarding the former, the *qsar* encodings extraordinary performance for some of the *hiv* datasets (see Figure 3) could be due to overfitting, owing to very similar sequences (see Supplementary Figure S2). With this respect, the *hiv_v3* dataset verifies this hypothesis further, as it contains very similar sequences and almost all encodings demonstrate very high performance on this dataset. In addition, the sequence embedding, shown in Supplementary Figure S2, provides a further, visual explanation. Finally, a low inter class similarity affects the class separation positively, which can also be observed by considering solely the performance of the amino composition encoding (see Supplementary Figure S3).

Albeit no real pattern emerges on the performance within biomedical domains one can still observe slightly similar results on these datasets (see Supplementary Figure S1), presumably owing to redundant sequences. We collected the datasets as they are and many studies build upon each other, which explains overlapping sequences in some cases.

As mentioned above, the class imbalance as well as the encoding type contribute mainly to the encoding performance. This explains also the result of the clustering, i.e. two major clusters for datasets and encoding groups. An exception refers to the *ksctriad* encoding, which clusters adjacent to StBEs, likely due to missing values (NA, see Figure 4) since for too short sequences this encoding type cannot be calculated. In addition, despite similar performance, it is not possible to draw conclusions on a similar function of the encodings. Far more datasets of the same biomedical application would have been necessary.

The similar performance of within-group encodings can be explained by adjacent parameter configurations, for example, a slightly larger gap length or window size (see Supplementary Figure S5), probably leading to only a marginal information change. Moreover, this observation supports our conducted *psekraac_* filtering, since it is likely that many of these encodings would perform similar, which in turn question the necessity of computing all of them.

### Similarity

The parameter configuration space for encodings emerging from the same group could also explain the similarity of the classifier outputs. That is, adjacent parameters provide no further or new insights for the machine learning model. This is in accordance with Kuncheva *et al.* (2003), who stated that diversity is a crucial condition for effective ensemble learning by mutually compensating weaknesses of single models (31). Certainly, this would not be possible if the classifier output is too similar. This is also the reason to consider SeBEs and StBEs, which show continuously low similarity (see Supplementary Figure S8) but also satisfying performance (see Figure 4). However, we observed, that the diversity cannot be arbitrarily high, since a greater diversity does not necessarily imply an improved class separation (see supplementary Figure S9).

The general trend, i.e. encodings from the same group show similar performance and lead to similar predictions can be verified by the statistical assessment, ultimately revealing a great variety of non-significant differences (see Supplementary Figure S11). The dataset correlation supports these observation impressively (see Supplementary Figure S5). The exceptions are the *pskraac_* and the *ngram_* encoding group, which is due to different sub-types, intrinsically generating different, within-group encodings.

### Time versus performance

The total computing time depends on the dataset size, i.e. the more sequences, the longer the required computation (see Supplementary Figure S12). A more detailed look at the total amount of sequences per dataset indicates that the computation time depends on the dataset size (see Supplementary Figure S12). However, the mean sequence length does not necessarily lead to an increased calculation time (see Supplementary Figure S12).

Moreover, some of the encodings impact the duration crucially, above all the StBEs (see Supplementary Figure S13). One can observe, that the majority of the SeBEs require less computation time and demonstrate at the same time a higher performance. We added the elapsed time required for the tertiary structure approximation to the total computation time of StBEs; thus, the calculation of the latter is in general prolonged. In addition, the tertiary structure approximation and the associated *electrostatic_hull* encoding as well as the *cgr* and *fldpc_* encoding, and finally the *psekraac_* filtering are main contributors to the total run time (see Supplementary Figure S13).

### Encoding recommendation

The recommendations serve as a general guideline, i.e. researchers have to decide case-wise, which encodings to use in particular. Some of the encodings seem to be redundant and usage is not reasonable at the first glance. However, using ensemble methods could compensate for weaknesses of single encodings, thus, even those encodings are applicable. This is also a matter of the dataset size and available resources. Moreover, although some encodings seem to work on imbalanced datasets, more research is necessary to draw meaningful conclusions.

## CONCLUSION

Our study marks the first comprehensive benchmark on various peptide encodings and we demonstrated, that in general, the performance of all encodings is similar and more or less independent from the biomedical task at hand. This allows us to reduce the vast number of encodings dramatically, paving the way for more sophisticated optimization methods in the future. A potential application refers to automated ensemble classifier configuration or to extend established automated machine learning methods like *auto-sklearn* (44). With this respect, a challenge remains the continuous search space, which could be tackled with pre-computed diversity measures to transform categorical hyperparameters (encodings) into numerical ones. Additional research is also necessary to verify whether and how StBEs can exhaust their full potential as part of ensemble classifiers. However, datasets with many sequences aligning to disordered regions can decrease the usability of StBEs clearly.

Our reproducible, parallelized pipeline conducts different analyses in order to get an expressive picture of the encoding performance across multiple biomedical domains. The results are aggregated across multiple biomedical domains and revamped as part of a great variety of interactive visualizations. All standardized datasets are available for download to comply with FAIR standards. The PEPTIDE REACToR allows researchers not only comparison at one glance, but also provides the state of the art for future encoding benchmarks, bundled in a single platform. With this respect, an extension is conceivable in order to allow researchers to upload their own (private) datasets.

## DATA AVAILABILITY

The results can be interactively accessed at https://peptidereactor.mathematik.uni-marburg.de/. The source code is available at https://github.com/spaenigs/peptidereactor. Due to the large size, intermediate data as well as intermediate results are available upon request.

## Supplementary Material

lqab039_Supplemental_File
