# Supplementary material for: A large-scale comparative study on peptide encodings for biomedical classification
Source: NAR Genom Bioinform. 2021 May 22;3(2):lqab039. doi: 10.1093/nargab/lqab039 (PMC8140742; doi:10.1093/nargab/lqab039)
Supplement: lqab039_Supplemental_File [file lqab039_supplemental_file.pdf]

Supplementary Note 1

Delaunay triangulation

Based on a set of nodes in a three-dimensional space, the Delaunay triangulation (DT), tries to find quadruples, fulfilling a specific condition: no other point must be within the circumscribed sphere of the tetrahedron, which is defined by a specific quadruple. If the condition is met, all nodes of the quadruple will be connected via edges. Finally, only these nodes are connected, fulfilling the criteria [1, 2].

First, we calculate the DT for each structure s_i using SciPy v1.3.1 [3]. Afterwards, we compute the adjacency matrix m of size 20×20 (the number of natural amino acids). An edge e in this graph is denoted as (a_j, a_k), i.e, the pair of amino acids, connected via the edge e. Since multiple occurrences of e with different distances between a_j and a_k are possible, in total five aggregation functions were implemented: average distance (average distance of equal edges), total distance (total sum of distances of equal edges), number of instances (the count of equal edges), frequency of instances (the frequency of equal edges), as well as cartesian product (the cartesian product between average distances and number of instances) [1]. In this study, we excluded edges, where two amino acids, a_j and a_k, are more than 15 Å apart. This value has been chosen, owing to the fact, that most of the distances were below 15 Å and due to an average distance of 10 Å of all edges.

Distance distribution

This StBE quantifies the distribution of distances between amino acid pairs a_j and a_k with certain functional types, i.e, chemical properties [4, 2], namely hydrogen bond acceptor, hydrogen bond donor, pi stacking centers, aliphatic and ambivalent donor-acceptor [4]. Note, that one amino acid could have more than one functional type. For each combination a_j, a_k and every functional type, one calculates the euclidean distance. Subsequently, we estimated the distribution with the kernel density estimator f, using a bandwidth of $h = 1$ and a Gaussian kernel K, denoted as

$$f = \frac{1}{nh} \sum_{j=1}^{n} K\left(\frac{t - x_j}{h}\right) \tag{1}$$

$$K(t) = \frac{1}{\sqrt{2\pi}} \exp\left(-\frac{1}{2}t^2\right) \tag{2}$$

and evaluated a fixed set of points on f and append it to the final feature vector [4, 5]. Specifically, we employed the function *gaussian_kde* provided by the SciPy package [3].

Distance frequency

For each input sequence s_i, the algorithm first replaces each amino acid a_k with its respective chemical group, that is, basic, hydrophobic, or others. Afterwards, s_i is split into three parts: N-terminal, middle section and C-terminal [6]. Note, that owing to various sequence lengths, different split methods are used. Refer to the original publication for the algorithmic details [6]. For each section, the distances are obtained by counting the number of amino acids between two, for instance, basic amino acids. Afterwards, the values are assigned to a distance class, hence distance frequencies, for each property group. Besides the distance frequencies for each group and part, also the amino acid, as well as the di-peptide composition, build up the final feature vector [6].

Electrostatic hull

A further StBE is the electrostatic hull (EH). First, the structure is optimized using the *amber* force field and is standardized for further processing by employing the PDB2PQR v2.1.1 [7] command-line tool. Afterwards, the solvent accessible surface (SAS) as well as the electrostatic potential (EP) is calculated by means of the APBS v1.5 [8] package. The coordinates of the EH are now computed based on the SAS. For the final feature vector, only these points from the hull are retained, where an EP has been determined in the previous step. Since the sequence length can vary, a cubic spline interpolation to the median length is conducted afterwards, utilizing the Interpol v1.3.1 package [9]. The general workflow as well as the core algorithm has been adapted from Löchel *et al.* (2018) [10].

Fourier transform

The Fourier transform (FT) SeBE, decomposes a continuous-valued input signal into its frequency domain, such that previously unknown patterns might be observable [2]. We leverage this circumstance by computing the discrete FT on $\hat{s}_i$. Specifically, a mapping $a_k \mapsto \hat{a}_k$ is obtained from the AAindex database [11]. Nagarajan *et al.* (2006) applied this encoding to predict antimicrobial activity [12].

Five level dipeptide compostion

The five-level di-peptide composition (FLDPC) SeBE [13], is based on five groups, i.e., the highest, high, medium, low, and lowest values of a specific amino acid index [11]. The assignment of an amino acid to a group occurs by employing the *k-Means* clustering algorithm, with $k = 5$. The final feature vector $\hat{s}_i$ for an input sequence s_i is composed of the sums of the frequencies of all di-peptides mapped to the same group [13].

Five level grouped composition

Exactly like the FLDPC, the five-level grouped composition (FLGC) is based on the five groups obtained from a specific amino acid index [11]. In order to compose the final feature vector, the amino acid composition is calculated for each sequence s_i and the frequencies of all amino acids from the same group are added up [13].

N-gram

The n-gram encoding [14] encodes sequences based on the singular value decomposition (SVD). There are several types of this encoding, depending on the according grouping of a specific amino acid: the di-peptide or tri-peptide composition (A), the exchange (E) as well as the structural groups (S). The latter encompasses amino acids, which have a tendency of an internal, ambivalent, or external configuration in the three-dimensional conformation of a protein [14] and the E group refers to six amino acids groups, computed from point-accepted mutations (PAM) [14, 15]. Furthermore, as the name suggests, two different sizes of n are considered: two (bi-gram) and three (tri-gram).

For the E and S groups, the preprocessing is conducted as follows. First, the cartesian product of the groups, i.e., E×E is calculated. Next, for all amino acids $a_k \in s_i$, a_k is mapped to its respective group, leading to $\hat{a}_k$. Now we count the occurrences of a bi-gram $\hat{a}_j, \hat{a}_k$ or tri-gram $\hat{a}_j, \hat{a}_k, \hat{a}_l$ and compute the total frequency with respect to the amount of all possible combinations c_i [14].

The next step comprises the matrix factorization step, hence SVD, in the form of

$$X = T \cdot S_* \cdot P \tag{3}$$

In particular, X is the encoded dataset $\hat{D}_i$ of size $n \times m$, where n is the number of features and m is the number sequences. T is a matrix with the left singular components of size $n \times k$, S_* denotes a diagonal matrix of size $k \times k$ and P refers to a matrix with the right singular components of size $k \times m$ [14]. Hereinafter, the SVD is employed as a feature reduction method, that is, the input feature space will be reduced from a n-dimensional space into a k-dimensional space. Thus, the transpose of P, hence P^T, is used subsequently as the final feature matrix [14]. For predicting unknown sequences X_u, the n-gram encoding requires to retain the matrices T and S_*. In the case of a prediction, the former and the inverse of the latter is utilized to scale the unknown data X_u into the same feature space:

$$P_u = X_u^T \cdot T \cdot S_*^{-1} \tag{4}$$

whereby P_u, the encoded matrix, has k columns as well as m_u rows and X_u^T, the transpose of the non-classified input data, is of size $m_u \times n$.

Quantitative structure-activity relationship

The quantitative structure-activity relationship (QSAR) StBE, an encoding type relating molecular properties of the structure to a certain activity, e.g., antimicrobial efficiency, has been added [2]. In particular, we adopted the QSAR-pipeline suggested by Haney *et al.* (2018) [16]. On each sequence s_i, a sliding window approach is applied, using a window size of k, if $|s_i| \geq k$. For each window w_l, we construct a molecule from the respective structure section utilizing RDKit v2020.03.4 (http://www.rdkit.org/). Note, that if $|s_i| < k$, the complete sequence will be used instead. In the present study we set $k = 20$. For each molecule, we used the Mordred v1.2.0 package, in order to calculate all molecular descriptors [17]. For a comprehensive descriptor list, refer to the original publication [17]. If $|s_i| < k$, the descriptor vector v_l will be used as feature vector as it is, otherwise the column-wise average will be used.

Weighted amino acid composition

The weighted amino acid composition [13] SeBE weights the respective amino acid composition aac of an amino acid a_i in a sequence s_i with the accompanying amino acid index [11] $f : a_i \mapsto \hat{a}_i : aac * f(a_i)$.

Supplementary Note 2

$$\text{Precision} = \frac{TP}{TP + FP} \tag{5}$$

$$\text{Recall} = \text{Sensitivity} = \frac{TP}{TP + FN} \tag{6}$$

$$\text{F1-score} = 2 \cdot \frac{\text{Precision} \cdot \text{Recall}}{\text{Precision} + \text{Recall}} \tag{7}$$

$$\text{Specificity} = \frac{TN}{TN + FP} \tag{8}$$

$$\text{MCC} = \phi = \frac{TP \times TN - FP \times FN}{\sqrt{(TP + FP)(TP + FN)(TN + FP)(TN + FN)}} \tag{9}$$

with TP = true positives, TN = true negatives, FP = false positives, FN = false negatives, MCC = Matthews Correlation Coefficient, and ϕ being the Phi coefficient.

Supplementary Note 3

The final report is composed of three sections, namely *Home*, *Single dataset*, and *Multiple datasets*, which fulfill specific, analytical purposes. The first provides a general overview of the study and the data, the second sheds light on the performance across multiple datasets, and the third section introduces the results for specific datasets. In general, all visualizations are interactive, hence including different mouse events (mouse-over, click, double-click, and scrolling). We used the *streamlit* v0.70.0 (https://www.streamlit.io/) framework to embed the graphics. Hereinafter, the respective visualizations are described in more detail.

Home

For the overview figure, the computation time vs. dataset size scatter plot (top-left) is connected with the t-SNE based sequence embedding (top-right). In addition, it provides a small description of the dataset and the link to the original publication (center). For download, a further link forwards the user to the location of prepared data (bottom). For better separation, the scatter plot uses logarithmic axes. The dataset name indicate where these data visualizations can be found at https://peptidereactor.mathematik.uni-marburg.de/.

Multiple datasets

The *Multiple dataset* section includes the *Overview*, the *Ranks*, *Clustering*, *Embedding*, and *Time* visualization, in order to investigate the performance all datasets

- *Overview.* The overview plot shows the performance of all encoding groups across all datasets. The figure is divided into two heatmaps. The upper one focuses on the grade of dataset imbalance compared to sequence- and structure-based encodings (SeBEs and StBEs, respectively) and the bottom one puts emphasis on the biomedical domain, i.e., the datasets are sorted accordingly. The respective groups are visually separated by horizontal and vertical bars. For the the top figure, a ratio of 0.35, i.e., positive : negative class, has been used for separation.

- *Ranks.* This figure visualizes the encoding performance as ranks across all datasets [18]. The encodings are grouped by SeBEs and StBEs. In addition, the datasets are sorted by imbalance. The respective groups are visually separated by horizontal and vertical bars.

- *Clustering.* The result of the automated clustering is shown here. Encoding groups and datasets are arranged according to the hierarchical clustering, further highlighted by row and column dendrograms.

- *Embedding.* The t-SNE based embedding of the sequences of the positive class. All datasets are arranged as a 3×4 scatter plot matrix. All sub-plots are sorted by cluster area ascending. In

addition, using the menu in the bottom-left allows to display additional datasets with a higher cluster area.

- *Time.* The total computation time of all datasets is visualized as a bar plot (top). Moreover, the scatter plot compares the computation time with the dataset size (bottom-left) and a further scatter plot relates the mean sequence length with the overall computing time (bottom-right).

Single dataset

More detailed information about specific datasets and the respective performance of all encodings reports the *Single dataset* section. It includes the *Overview*, *Metrics*, *Curves*, *Similarity*, *Diversity*, *Difference*, *Composition*, *Correlation*, as well as the *Time* sub-sections.

- *Overview.* This section summarizes the CV runs by means of the median of all splits and the resulting overall median. The encodings are grouped into sequence- and structure-based encodings (SeBEs and StBEs, respectively). Moreover, parameterized encodings have been aggregated into single groups. The number of encodings per group corresponds to the circle size. The range of medians per split and group is highlighted as a shaded area and the height of the line is determined from the best encoding per group.

- *Metrics.* The particular metrics are shown here. That is, the left chart shows the median performance across all cross-validation splits of all encodings. In addition, the top 20 encodings are highlighted with 100 % opacity. For more fine-grained insights, the CV results of the top encodings are shown in the box plot (right).

- *Curves.* Receiver-operation characteristic (ROC) as well as the Precision-Recall (PR) curves are plotted are visualized in this section. The top row shows the respective curves for the overall top 6 encodings, whereas the bottom row shows the top 3 SeBEs and StBEs, respectively.

- *Similarity.* The similarity of encodings is visualized as heat maps, with the Phi correlation ϕ being visualized in the top row and the disagreement measure D is visualized in the bottom row. For the latter, higher values indicate a greater diversity, whereas lower values are preferred for the Phi correlation.

- *Diversity.* The next section contains the pairwise similarity of selected encodings. The x- and the y-axis are showing the predicted probabilities of the classifiers trained with the respective encodings picked due to their grade of disagreement. The left column shows encodings with a high diversity and the right column visualizes the encodings with the best single performance. The middle section, i.e., from the second to the fourth panel, shows encodings with an ascending disagreement. Moreover,

the top row compares sequence- and structure-based encodings and the bottom row all versus all encodings. In addition, the cluster quality is denoted as the Davis-Bouldin score, where lower values indicate a better separation of the clusters [19].

- *Difference* (CD). A statistical comparison of all encodings can be found in the CD section. Critical, i.e., significantly, different classifiers are colored in black. The heat map shows the respective CDs for all encodings. The right bar chart counts the critical different encodings per group and the lower part converts the heat map data into a one-dimensional space by grouping encodings according to the CD. The circle size denotes the number of encodings per group.
- *Composition.* Meta information about the dataset at hand is visualized in this section. The top chart shows the overall class distribution. The positive class is green colored and the negative class accordingly purple. The plot in the middle visualizes the number of sequences per length for the respective class. Finally, the bottom row shows the relative composition of amino acids as a bar chart (left) and the sequence similarity based on a t-SNE embedding (right).
- *Correlation.* The dataset correlation is visualized as a circular dendrogram, which aggregates more related datasets into the same branches. The relatedness is based on adjusted RV-coefficient. Encodings from the same group are highlighted in the same color.
- *Time* This section deals with the required execution time of every task. In particular, the scatter charts compares the median performance of the encoding groups with their respective calculation time. The top-left corner shows the groups with the preferred properties, i.e., fast computation and high performance. Moreover, the scatter chart shows the execution time for each step on a log scale. Each meta node type is colored accordingly.

Supplementary Note 4

PseKRAAC encoding

This SeBE takes four parameters, leading to hundreds or even thousands, in general k's of encoded datasets $\{\hat{D}_{i_1}, \dots, \hat{D}_{i_k}\}$. In order to reduce this vast amount of data, i.e, to find a representative subset of encoded datasets $\Theta \cap \{\hat{D}_{i_1}, \dots, \hat{D}_{i_k}\}$, the filtering is conducted as follows: first, the datasets are grouped according to their descriptor type, i.e., for each $\hat{D}_{i_j} \in \Theta$ of the same descriptor type, the datasets are interpolated to the same dimension using a one-dimensional linear interpolation. The Pearson correlation coefficient R is calculated on the vectorized matrices $\hat{D}_{i_x}$ and $\hat{D}_{i_y}$, denoted as X and Y, respectively:

$$R_{X,Y} = \frac{\sum_{i=1}^{n}(X_i - \overline{X})(Y_i - \overline{Y})}{\sqrt{\sum_{i=1}^{n}(X_i - \overline{X})^2 \sum_{i=1}^{n}(Y_i - \overline{Y})^2}} \tag{10}$$

n is the length of the vectorized matrices, X_i and Y_i are data points from the respective datasets X or Y and $\overline{X}$ as well as $\overline{Y}$ being the respective mean. Next, for each descriptor type and based on the Pearson correlation coefficients from the previous step, a distance matrix m is calculated. m is used as the pre-computed distance matrix for the successive t-distributed Stochastic Neighbor Embedding (t-SNE, default parameters) [20] in order to embed m into a two-dimensional space. Finally, the representative dataset for each of the 19 descriptor types (see Supplementary Table 4) is determined by computing the cluster center by means of the *k-Medoids* algorithm [21].

Amino acid index correlation

Some amino acid indices (AAI) are highly correlated [11]. Hence, let X be a matrix of size $20 \times k$ with 20 rows for the corresponding natural amino acids and k columns for each AAI. First, we computed the pairwise Pearson correlation coefficient for each column in X, i.e., the AAI, using Equation 10. Next, we utilized principal component analysis (PCA) [22] to compute the first principal component, that is, to reduce the size of X to a one-dimensional matrix $\hat{X}$. By regarding $\hat{X}$ as distances, we observed that AAIs with a high separation after PCA also have a high correlation and conversely, AAIs with a low separation after PCA also have a low correlation. Hence, we only keep those indices with a correlation closely at 0.0 ± 0.3. Finally, only those encoded datasets based on low correlated AAIs are used for the later benchmark.

Supplementary Figures

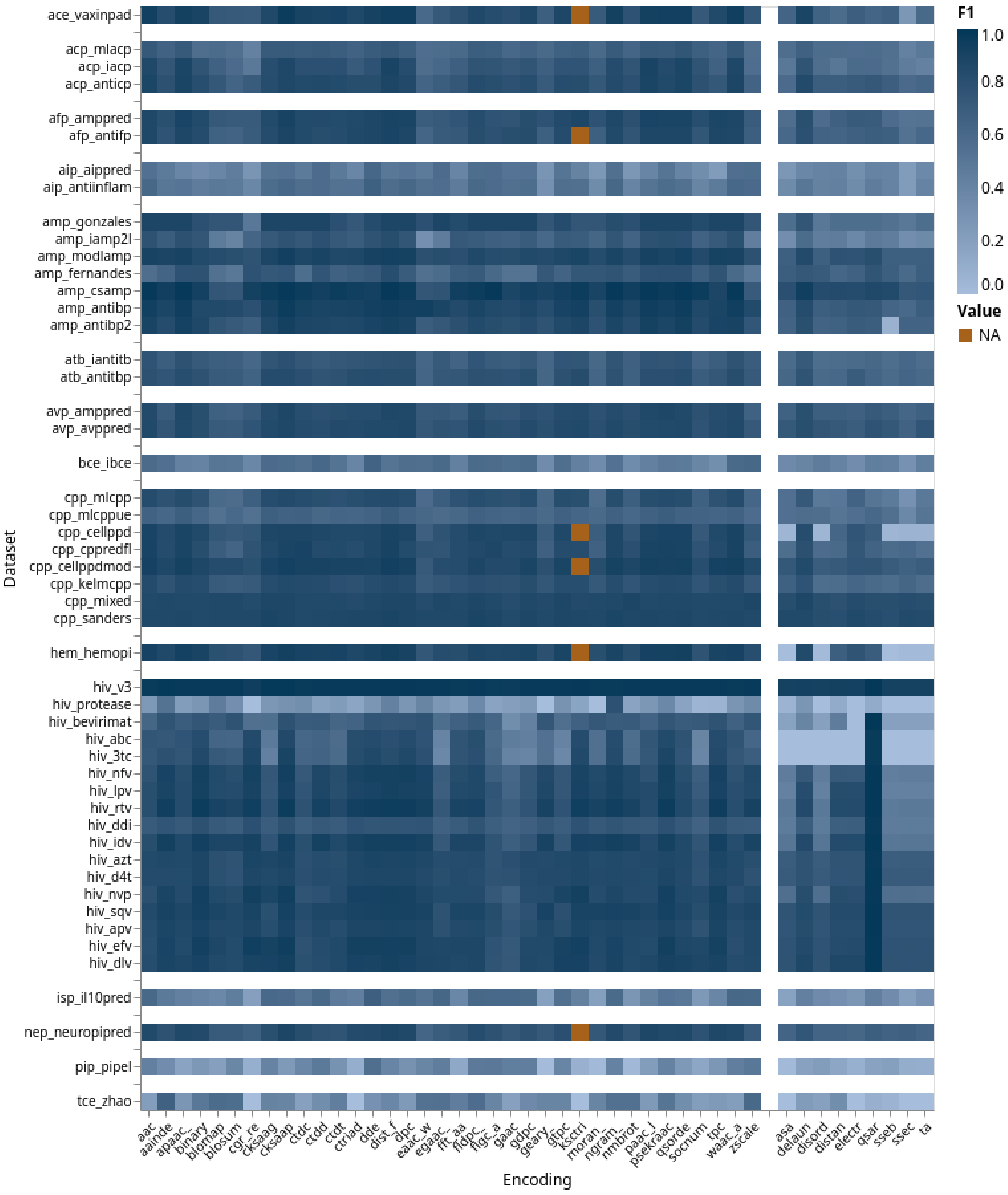

Supplementary Figure 1: **Encoding group performance, sorted by biomedical domains and encoding type.** Color coding corresponds to the max F1-score of a group. The x-axis is organized by sequence- and structure-based encodings. The y-axis is sorted by biomedical application. Groups are separated by gaps.

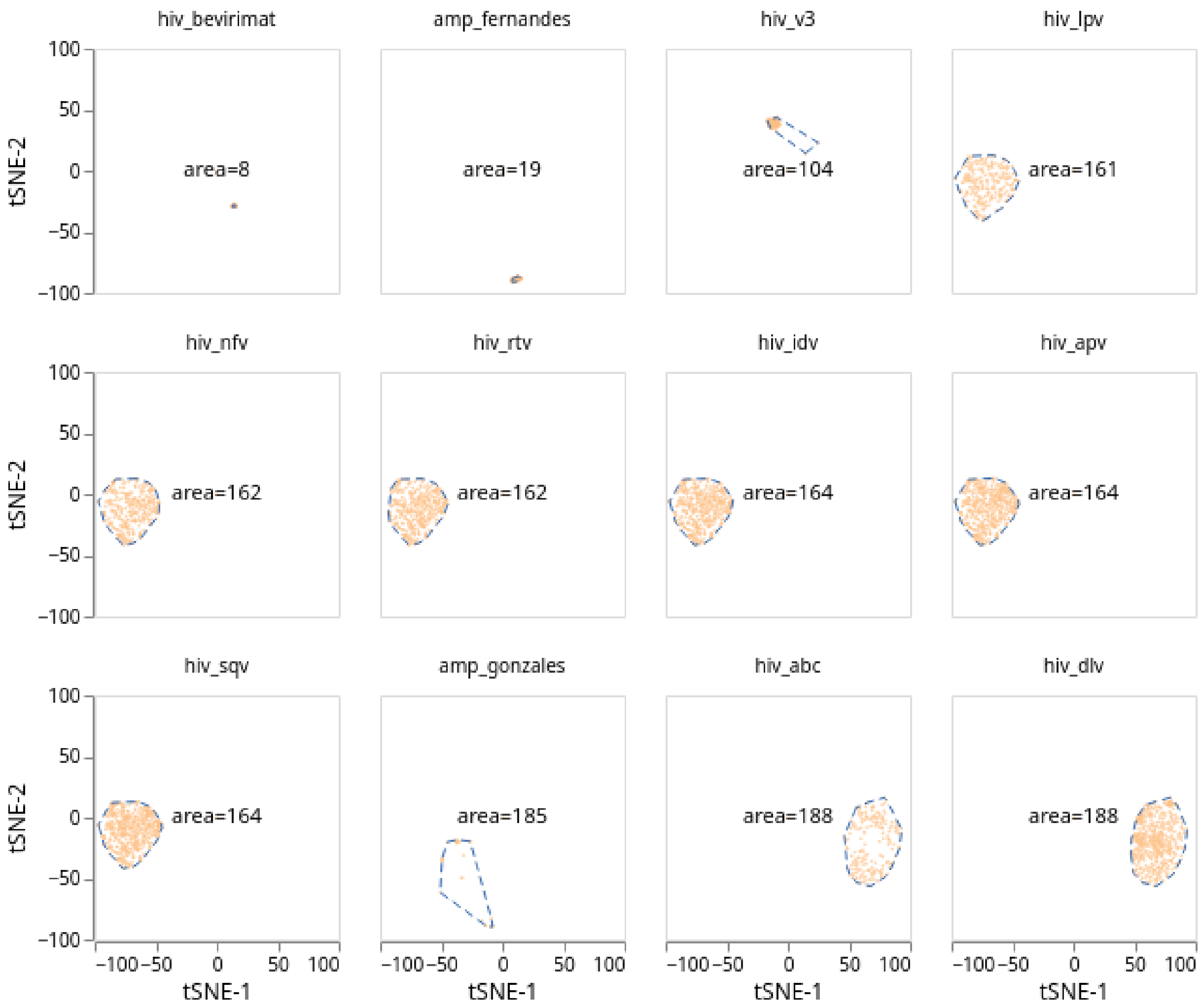

Supplementary Figure 2: **t-SNE-based embedding of sequences part of the positive class.** The ordering of datasets corresponds to the respective cluster area. Ranks 1 to 12 are shown.

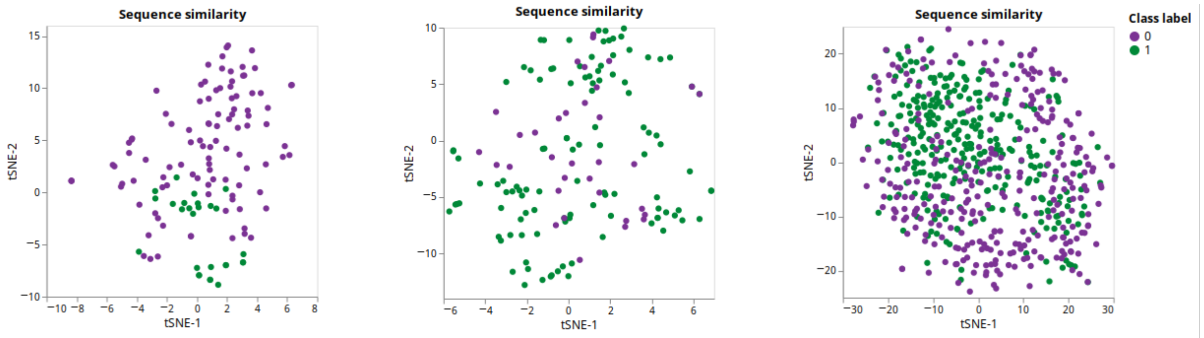

Supplementary Figure 3: **Sequence similarity based on the t-SNE embedding.** The embedding is based on the amino acid composition (AAC). The graphic shows exemplary the *amp_gonzales* (left), *cpp_sanders* (center), and *hiv_ddi* (right) datasets with a median F1-score for the AAC encoding of 0.89, 0.86, and 0.72, respectively.

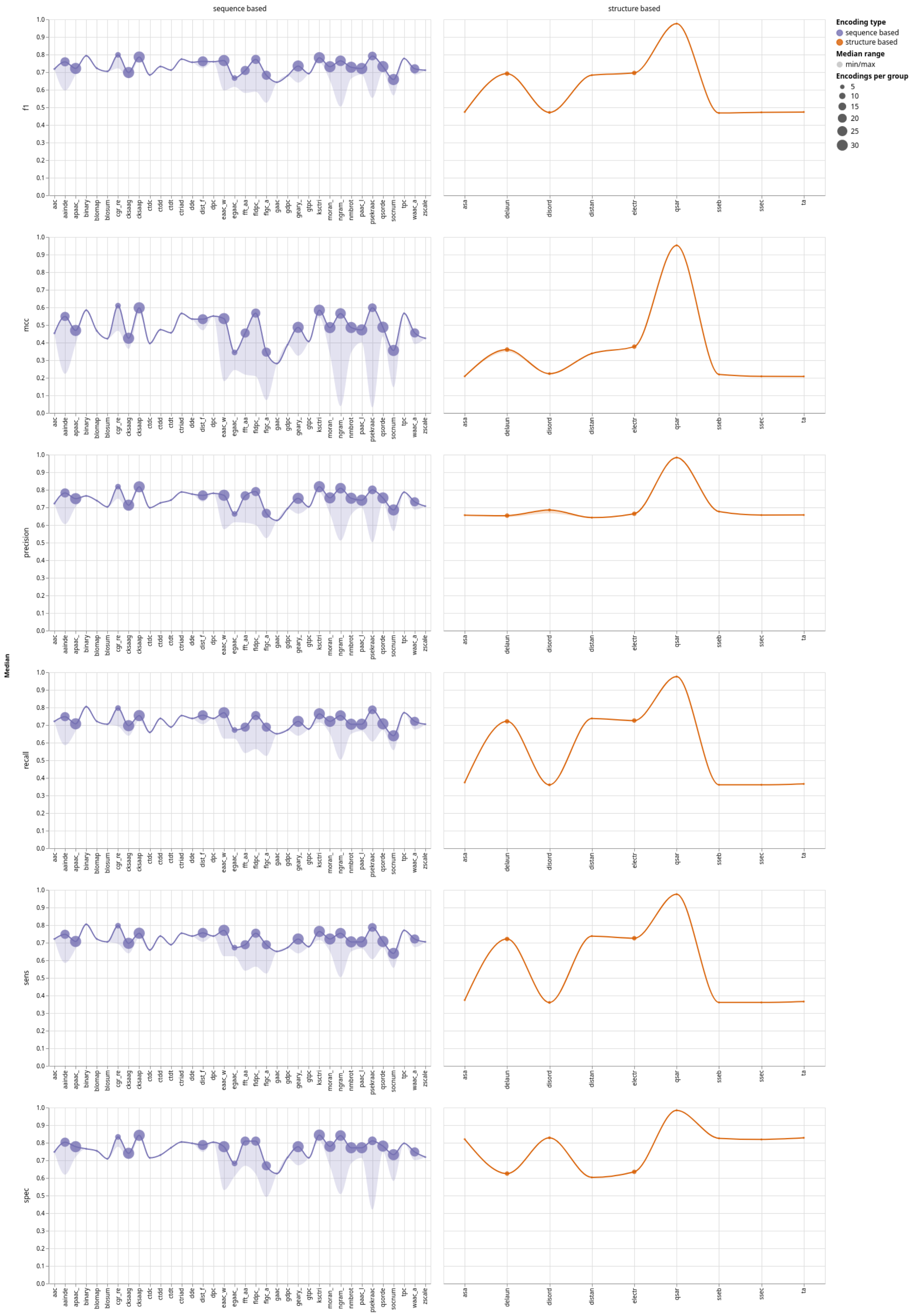

Supplementary Figure 4: **Median performance of encoding groups.** The min/max range is visualized by the shaded area and the circle size corresponds to the number of encodings per group. Circle heights depict scores of best performing encoding within a group. The graphic shows the example of the *hiv_ddi* dataset.

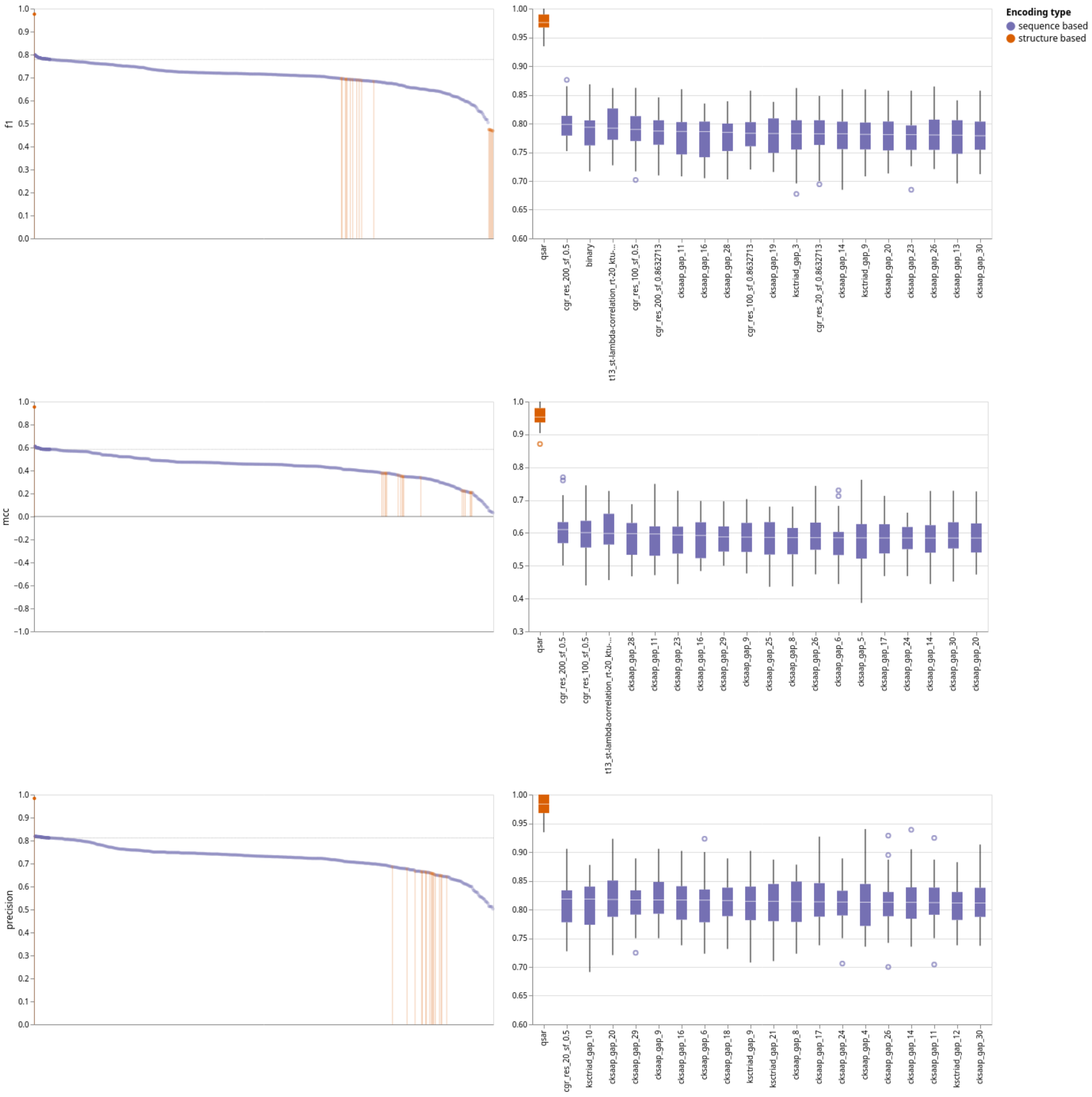

Supplementary Figure 5: **Detailed metrics and distribution for the F1-score, MCC, and Precision.** Detailed metrics for each encoding (left) including the F1-score, MCC, and Precision. The box plots on the right show the respective distribution of the repeated cross-validations. The graphic shows the example of the *hiv_ddi* dataset.

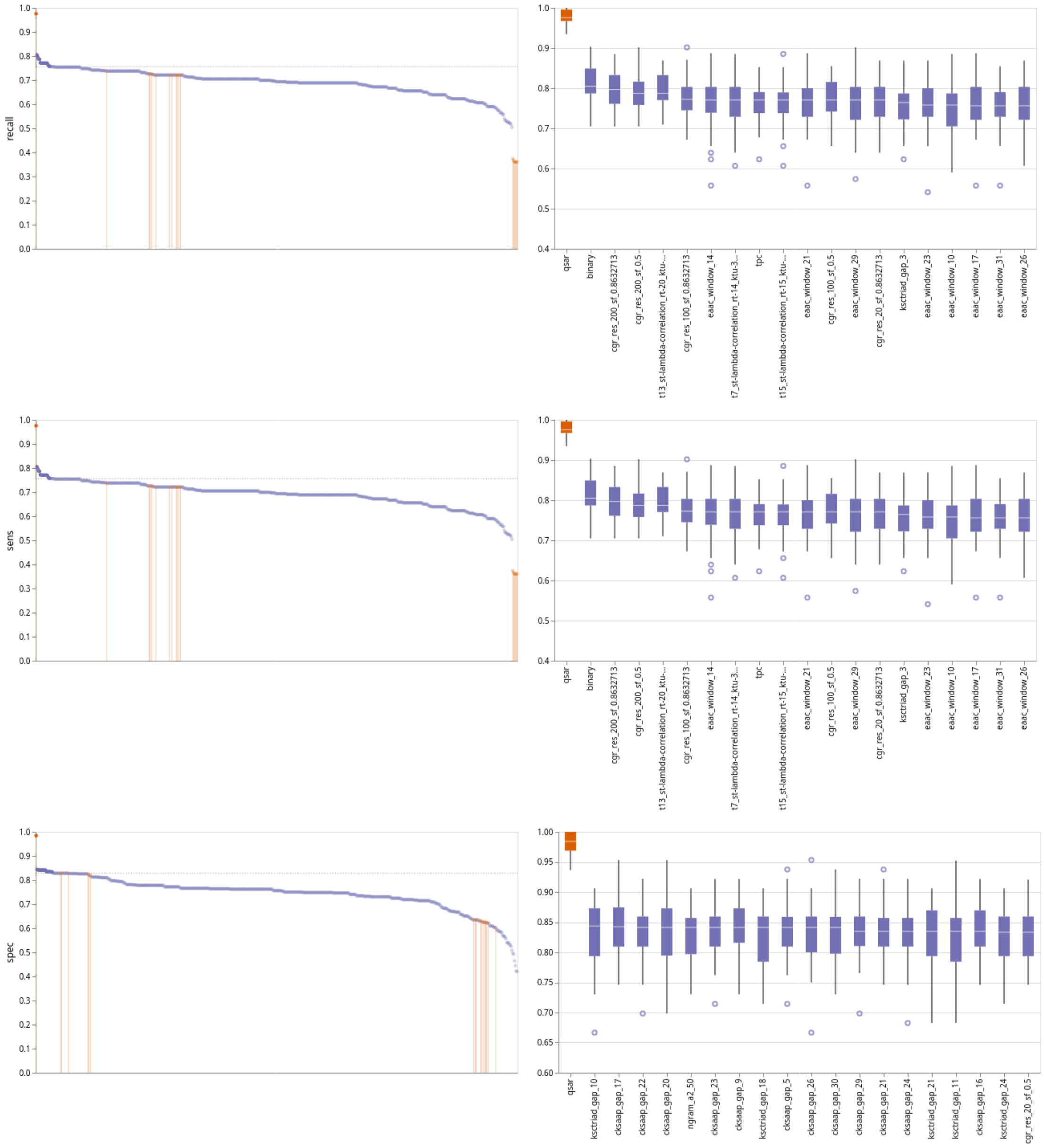

Supplementary Figure 6: **Detailed metrics and distribution for the Recall (Sensitivity) and Specificity.** Detailed metrics for each encoding (left) including the Recall (Sensitivity) and Specificity. The box plots on the right show the respective distribution of the repeated cross-validations. The graphic shows the example of the *hiv_ddi* dataset.

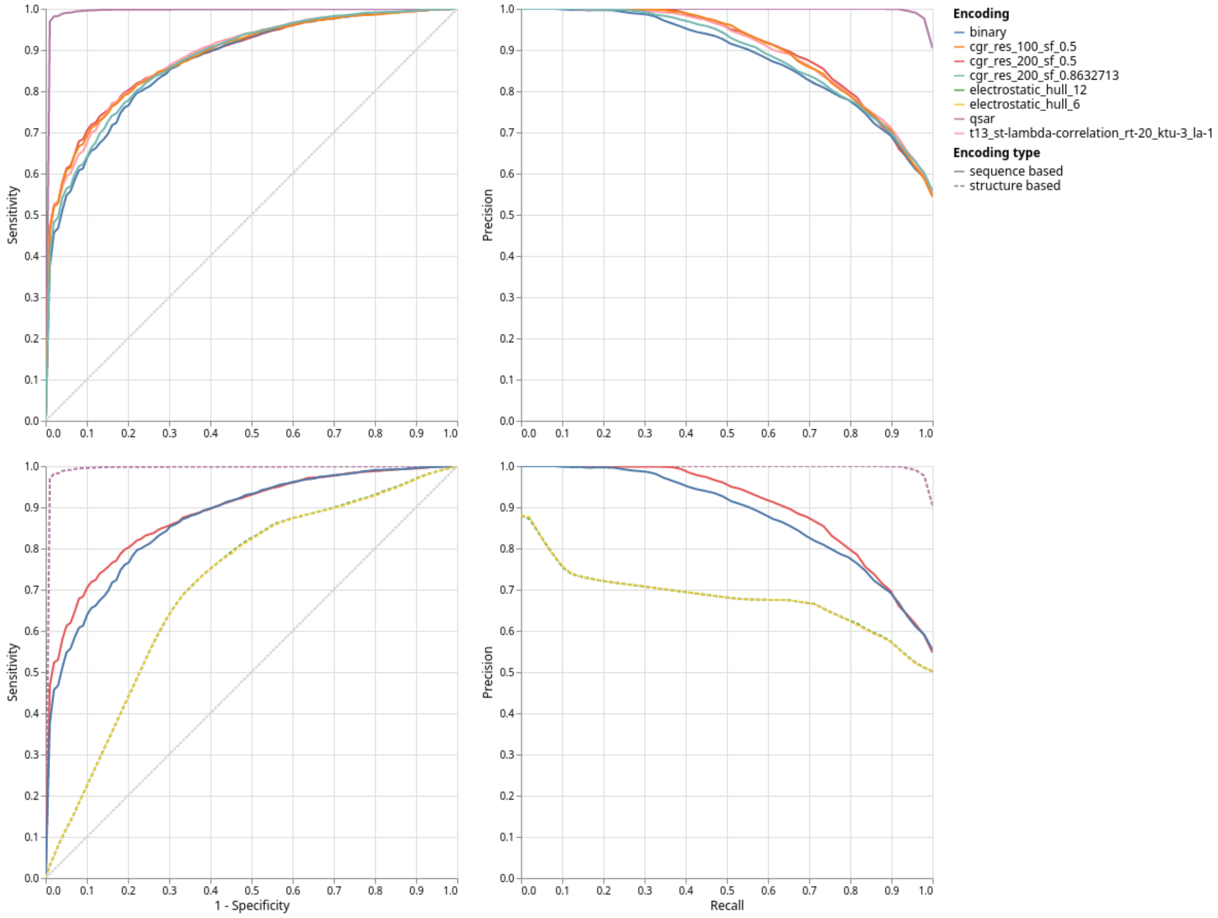

Supplementary Figure 7: **ROC and PR curves of the top encodings.** ROC (receiver operating characteristic, left) and PR curve (Precision/Recall, right) for top 6 encodings (top) and top 3 sequence- and top 3 structure-based encodings (bottom), based on the F1-score. The graphic shows the example of the *hiv_ddi* dataset.

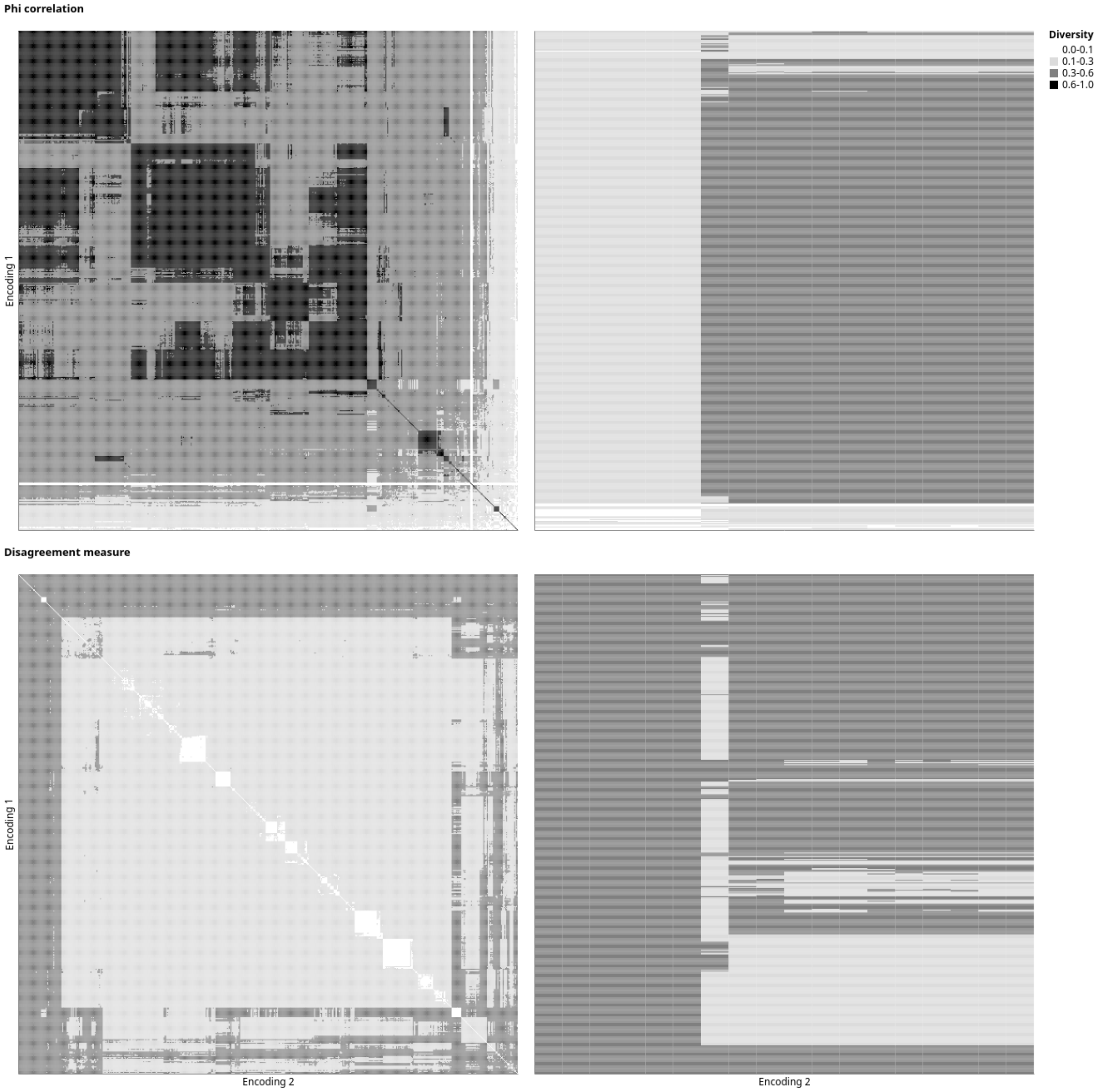

Supplementary Figure 8: **The similarity of classifier outputs.** Similarity of the classifier outputs between all encodings (left) and sequence- vs. structure-based encodings (right), based on correlation (top) and disagreement (bottom). The higher the diversity value, the higher the similarity (Phi correlation). Accordingly, the lower the diversity, the higher the similarity (Disagreement measure). The graphic shows the example of the *hiv_ddi* dataset.

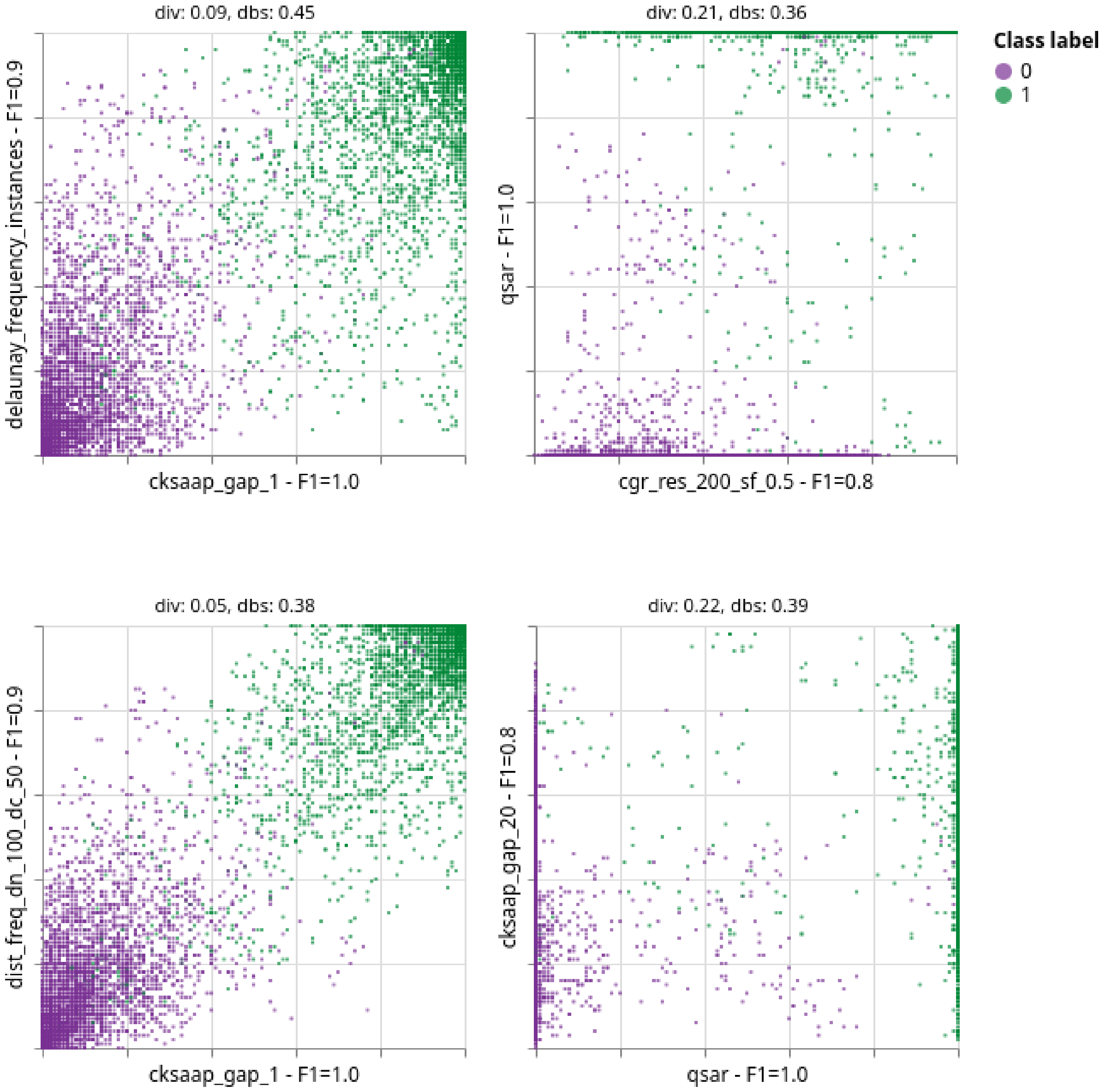

Supplementary Figure 9: **Pairwise predicted probabilities and class separation for two datasets.** Predicted probabilities for the respective class labels applied on the x- and y-axis. Encodings are selected with respect to their level of disagreement (div) and cluster quality, depicted as the Davis-Bouldin score (dbs, lower is better). The graphic shows the example of the *ace_vaxinpad* (left) and the *hiv_ddi* dataset (right) for sequence- vs. structure-based encodings (top) and all vs. all encodings (bottom).

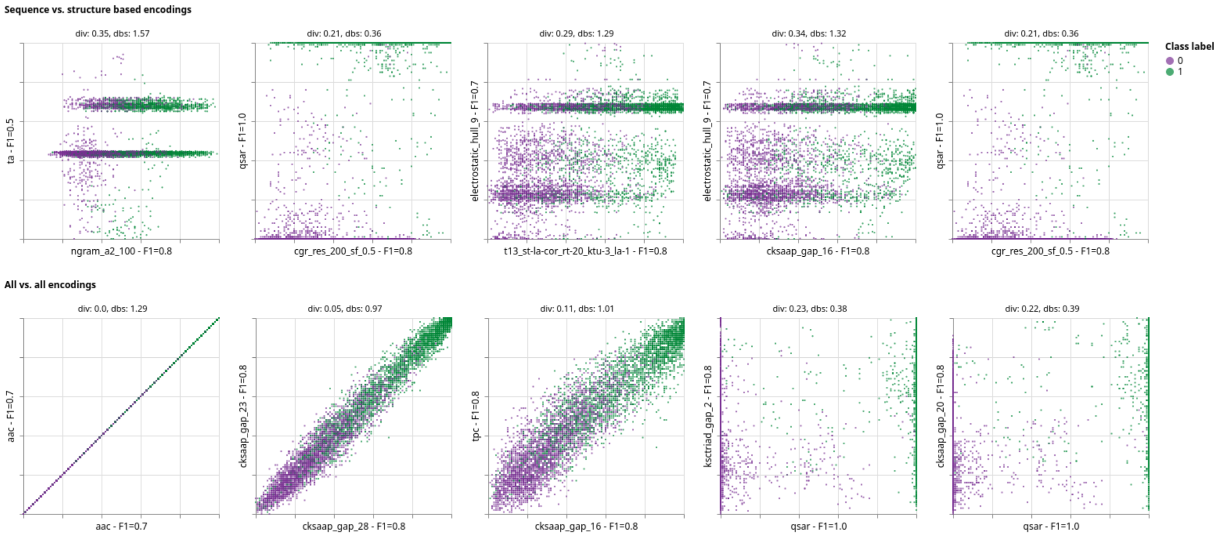

Supplementary Figure 10: **Pairwise predicted probabilities and class separation.** Predicted probabilities for the respective class labels applied on the x- and y-axis. Encodings are selected with respect to their level of disagreement (div) and cluster quality, depicted as the Davis-Bouldin score (dbs, lower is better). The graphic shows the example of the *hiv_ddi* dataset for sequence vs. structure based encodings (top) and all vs. all encodings (bottom).

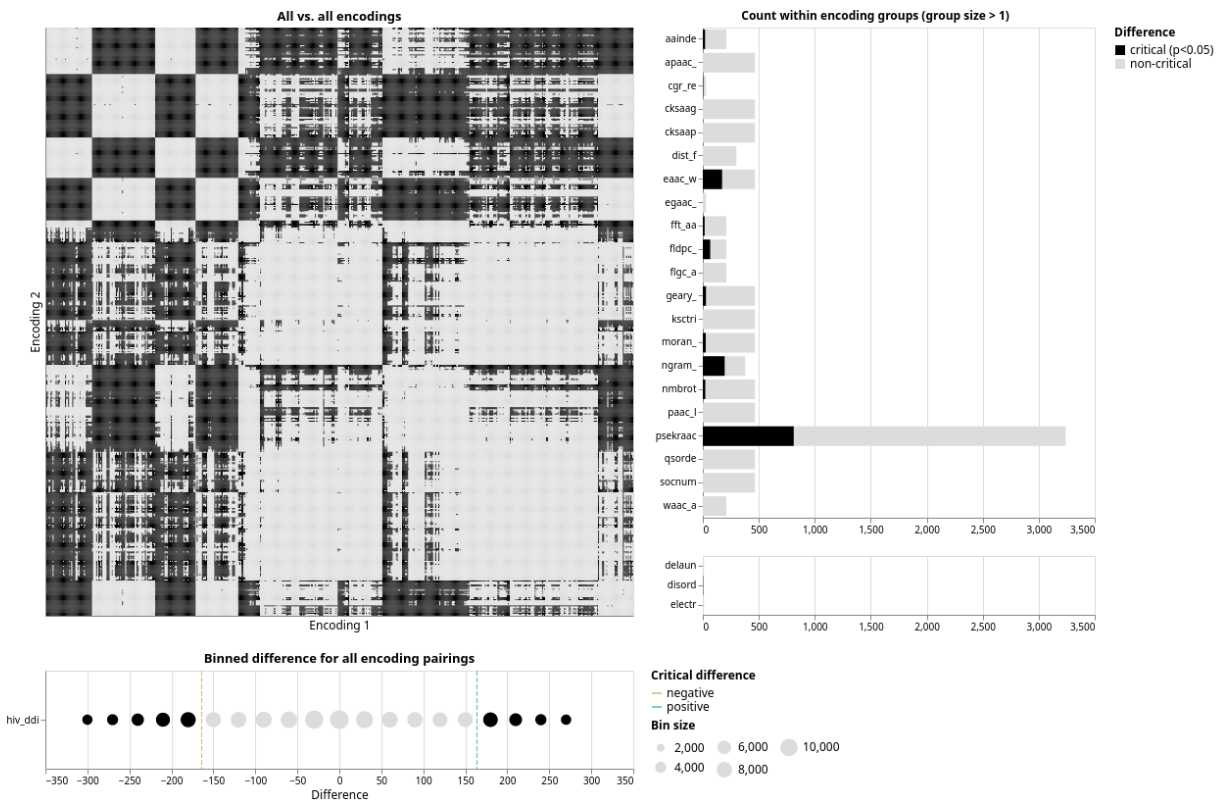

Supplementary Figure 11: **Statistical assessment of classifier outputs.** Statistical comparison (critical difference) of model performance per fold for all vs. all encodings (top-left), within an encoding group (top-right) and the binned counts distribution of differences for all comparisons (bottom-left). Black values refer to critical, hence statistically significant, different classifier outputs. The graphic shows the example of the *hiv_ddi* dataset.

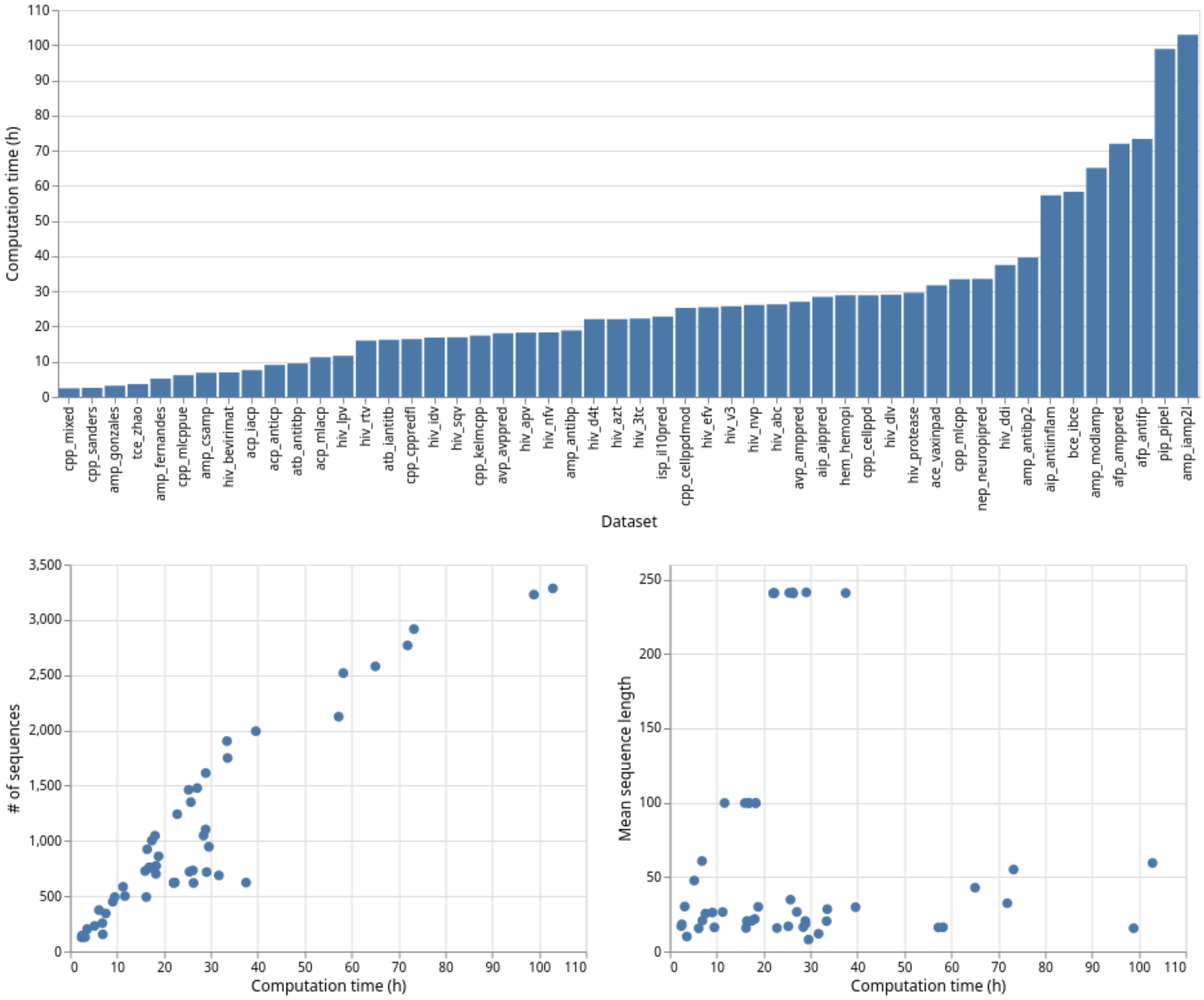

Supplementary Figure 12: **Total computation time.** Total computation time per dataset (top), the number of sequences vs. computation time (bottom-left), and mean sequence length per dataset vs. computation time (bottom-right).

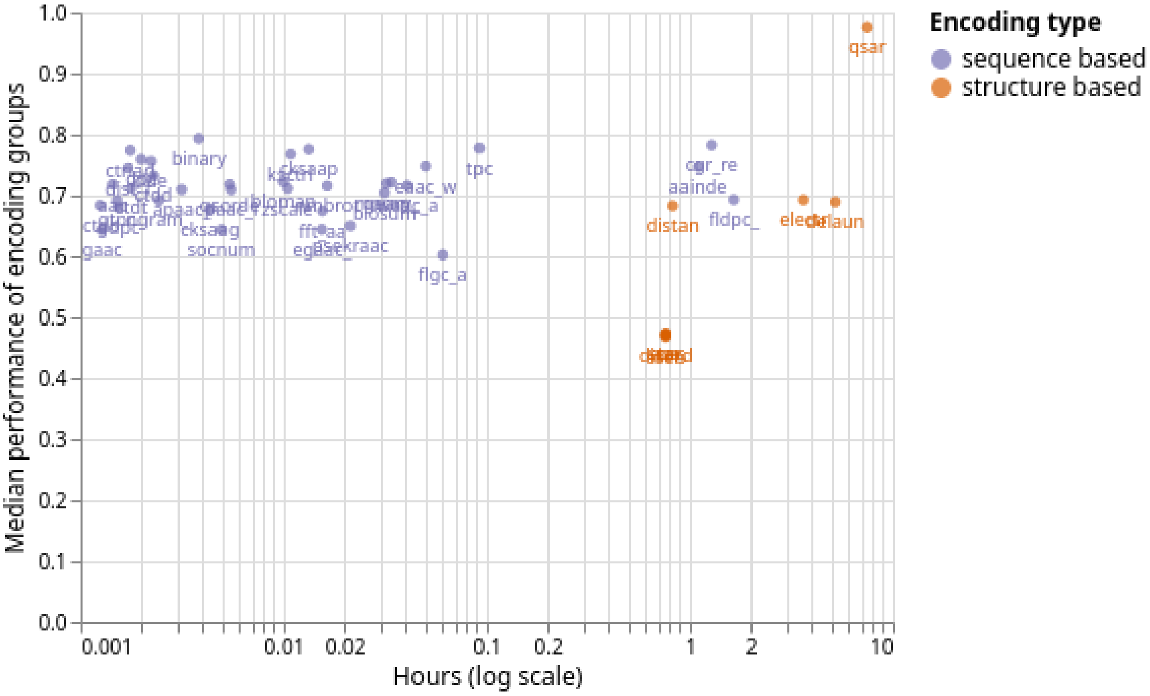

Supplementary Figure 13: **Encoding group performance vs. computation time.** Median of encoding group performance vs. elapsed time. The graphic shows the example of the *hiv_ddi* dataset.

Supplementary Tables

Supplementary Table 1: Datasets collected for this study. Biomedical applications are separated via a horizontal rule.

Dataset	Size	# Positive	# Negative	Reference
ace_vaxinpad	688	303	385	[23]
acp_anticp	450	225	225	[24]
acp_iacp	344	138	206	[25]
acp_mlacp	585	187	398	[26]
afp_amppred	2768	1384	1384	[27]
afp_antifp	2916	1459	1457	[28]
aip_aippred	1049	420	629	[29]
aip_antiinflam	2124	863	1261	[30]
amp_antibp	861	431	430	[31]
amp_antibp2	1993	999	994	[32]
amp_csamp	256	128	128	[33]
amp_fernandes	231	115	116	[34]
amp_gonzales	129	27	102	[35]
amp_iamp2l	3284	879	2405	[36]
amp_modlamp	2579	1225	1354	[37]
atb_antitbp	492	246	246	[38]
atb_iantitb	492	246	246	[39]
avp_amppred	1478	739	739	[27]
avp_avppred	1047	599	448	[40]
bce_ibce	2518	1110	1408	[41]
cpp_cellppd	1614	807	807	[42]
cpp_cellppdmod	1462	732	730	[43]
cpp_cppredfl	924	462	462	[44]
cpp_kelmcpp	1003	504	499	[45]
cpp_mixed	128	97	31	[46]
cpp_mlcpp	1903	738	1165	[47]
cpp_mlcppue	374	187	187	[47]
cpp_sanders	145	111	34	[48]
hem_hemopi	1104	522	582	[49]
hiv_3tc	624	195	429	[50]
hiv_abc	619	179	440	[50]
hiv_apv	702	424	278	[50]
hiv_azt	621	322	299	[50]
hiv_bevirimat	155	43	112	[51]
hiv_d4t	621	336	285	[50]
hiv_ddi	623	306	317	[50]
hiv_dlv	718	455	263	[50]
hiv_efv	721	447	274	[50]
hiv_idv	758	384	374	[50]
hiv_lpv	501	223	278	[50]
hiv_nfv	775	303	472	[50]
hiv_nvp	733	415	318	[50]
hiv_protease	947	149	798	[52]
hiv_rtv	728	349	379	[50]
hiv_sqv	761	457	304	[50]
hiv_v3	1351	200	1151	[53]
isp_il10pred	1242	394	848	[54]
nep_neuropipred	1750	875	875	[55]
pip_pipel	3228	833	2395	[56]
tce_zhao	203	36	167	[57]

Supplementary Table 2: Descriptive statistics on the datasets used in this study. s^+ and s^-: sequences of the positive and the negative class, respectively. $[s^+]$ and $[s^-]$: length interval, i.e., min and max sequence length per class. $\overline{s}$ and $\widetilde{s}$: mean and median of the respective class. Also refer to https://peptidereactor.mathematik.uni-marburg.de/ for according visualizations.

**Dataset**	**# (s^+, s^-)**	$[s^+]$	$[s^-]$	$\overline{s^+}$, **± std**	$\overline{s^-}$, **± std**	$\widetilde{s^+}$	$\widetilde{s^-}$
ace_vaxinpad	303, 385	3, 30	4, 30	11.01, 6.47	12.63, 7.33	9.0	12.0
acp_anticp	225, 225	5, 113	5, 54	23.51, 14.68	28.86, 13.94	21.0	30.0
acp_iacp	138, 206	11, 97	11, 39	25.91, 14.44	25.2, 7.1	24.0	25.0
acp_mlacp	185, 398	11, 47	11, 50	23.36, 8.91	27.96, 9.09	24.0	27.0
afp_amppred	1381, 1384	10, 255	10, 94	37.98, 33.97	26.9, 13.11	29.0	24.0
afp_antifp	1459, 1457	4, 100	4, 100	55.24, 25.06	54.93, 25.06	55.0	55.0
aip_aippred	420, 629	11, 25	11, 22	17.23, 2.94	15.79, 1.89	16.0	15.0
aip_antiinflam	863, 1261	8, 30	7, 30	16.36, 4.15	15.98, 2.86	16.0	15.0
amp_antibp	431, 430	30, 30	30, 30	30.0, 0.0	30.0, 0.0	30.0	30.0
amp_antibp2	981, 994	6, 94	6, 94	29.66, 13.78	29.74, 13.78	27.0	27.0
amp_csamp	121, 128	16, 79	28, 119	36.98, 9.74	84.77, 23.82	35.0	85.0
amp_fernandes	113, 116	12, 99	11, 100	36.56, 16.72	57.95, 23.26	35.0	54.0
amp_gonzales	27, 102	14, 37	11, 84	21.59, 4.48	32.44, 14.87	23.0	32.0
amp_iamp2l	878, 2405	5, 103	11, 100	31.98, 17.92	69.51, 25.31	29.0	76.0
amp_modlamp	1225, 1354	30, 100	30, 50	45.4, 17.82	40.53, 5.69	39.0	40.0
atb_antitbp	246, 246	5, 61	5, 60	15.83, 10.12	16.59, 10.28	12.0	13.0
atb_iantitb	246, 244	5, 61	5, 56	15.83, 10.12	15.69, 10.41	12.0	13.0
avp_amppred	738, 739	10, 255	10, 46	30.3, 29.25	22.9, 8.47	25.0	21.0
avp_avppred	599, 447	6, 107	6, 58	24.42, 10.62	18.23, 8.13	20.0	15.0
bce_ibce	1110, 1408	11, 24	11, 49	16.65, 3.21	15.92, 3.59	16.0	15.0
cpp_cellppd	807, 807	3, 61	5, 49	16.14, 6.98	21.06, 9.84	15.0	21.0
cpp_cellppdmod	563, 695	3, 38	3, 41	16.94, 7.3	17.04, 7.27	16.0	16.0
cpp_cppredfl	462, 462	10, 58	10, 61	20.52, 8.57	20.4, 8.43	18.0	18.0
cpp_kelmcpp	483, 499	11, 33	8, 49	17.93, 5.14	23.14, 7.95	17.0	23.0
cpp_mixed	94, 31	5, 38	7, 27	17.98, 6.31	14.58, 5.41	18.0	15.0
cpp_mlcpp	737, 1165	5, 48	5, 46	18.73, 8.11	21.41, 7.23	17.0	20.0
cpp_mlcppue	181, 187	5, 61	5, 36	15.75, 7.26	15.27, 6.13	15.0	15.0
cpp_sanders	111, 34	5, 43	8, 36	18.68, 6.84	16.91, 5.94	18.0	16.0
hem_hemopi	522, 582	5, 98	4, 86	20.21, 8.51	20.71, 10.55	18.0	18.0
hiv_3tc	195, 429	141, 249	170, 249	240.56, 7.79	241.0, 4.3	241.0	240.0
hiv_abc	179, 440	170, 249	31, 249	240.87, 5.65	240.4, 11.49	241.0	241.0
hiv_apv	423, 278	99, 107	99, 106	99.76, 1.35	99.68, 1.11	99.0	99.0
hiv_azt	322, 299	170, 249	141, 249	241.08, 4.39	240.65, 6.73	241.0	240.0
hiv_bevirimat	43, 112	19, 21	20, 21	20.53, 0.55	20.87, 0.34	21.0	21.0
hiv_d4t	336, 285	170, 249	31, 249	241.06, 4.29	239.91, 14.21	241.0	240.0
hiv_ddi	306, 317	170, 249	141, 249	240.92, 4.41	240.82, 6.6	240.0	241.0
hiv_dlv	455, 263	240, 253	170, 250	241.67, 2.19	240.66, 5.18	241.0	240.0
hiv_efv	447, 274	170, 253	74, 250	241.51, 4.03	240.14, 11.39	241.0	240.0
hiv_idv	382, 374	99, 107	99, 106	99.7, 1.27	99.72, 1.21	99.0	99.0
hiv_lpv	223, 278	99, 107	99, 106	99.68, 1.14	99.8, 1.29	99.0	99.0
hiv_nfv	300, 472	99, 107	99, 106	99.79, 1.35	99.64, 1.15	99.0	99.0
hiv_nvp	414, 318	240, 253	170, 250	241.72, 2.22	240.76, 4.77	241.0	240.0
hiv_protease	149, 798	8, 8	8, 8	8.0, 0.0	8.0, 0.0	8.0	8.0
hiv_rtv	348, 379	99, 107	99, 106	99.74, 1.29	99.73, 1.22	99.0	99.0
hiv_sqv	456, 304	99, 107	99, 106	99.71, 1.26	99.7, 1.21	99.0	99.0
hiv_v3	200, 1151	32, 37	32, 38	34.71, 0.94	34.86, 0.46	35.0	35.0
isp_il10pred	394, 848	8, 42	8, 27	16.8, 4.72	15.24, 1.52	15.0	15.0
nep_neuropipred	875, 875	4, 100	4, 100	28.19, 21.86	28.64, 21.81	20.0	20.0
pip_pipel	833, 2395	11, 25	11, 25	16.57, 2.78	15.3, 1.47	15.0	15.0

tce_zhao	36, 167	10, 10	10, 10	10.0, 0.0	10.0, 0.0	10.0	10.0

Supplementary Table 3: Description of datasets used in this study.

**Dataset**	**Description**	**Reference**
ace_vaxinpad	Prediction of peptides for modulating antigen presenting cells (modulating/non modulating).	[23]
acp_anticp	Prediction of peptides with cytotoxic efficiency against cancer cells (cytotoxic/non-cytotoxic).	[24]
acp_iacp	Prediction of peptides with cytotoxic efficiency against cancer cells (cytotoxic/non-cytotoxic).	[25]
acp_mlacp	Prediction of peptides with cytotoxic efficiency against cancer cells (cytotoxic/non-cytotoxic).	[26]
afp_amppred	Prediction of peptides with anti-fungal efficiency (anti-fungal/not anti-fungal).	[27]
afp_antifp	Prediction of peptides with anti-fungal efficiency (anti-fungal/not anti-fungal).	[28]
aip_aippred	Prediction of therapeutic peptides against inflammatory diseases (anti-inflammatory/not anti-inflammatory).	[29]
aip_antiinflam	Prediction of therapeutic peptides against inflammatory diseases (anti-inflammatory/not anti-inflammatory).	[30]
amp_antibp	Prediction of peptides with anti-microbial efficiency (anti-microbial/not anti-microbial).	[31]
amp_antibp2	Prediction of peptides with anti-microbial efficiency (anti-microbial/not anti-microbial).	[32]
amp_csamp	Prediction of peptides with anti-microbial efficiency (anti-microbial/not anti-microbial).	[33]
amp_fernandes	Prediction of peptides with anti-microbial efficiency (anti-microbial/not anti-microbial).	[34]
amp_gonzales	Prediction of peptides with anti-microbial efficiency (anti-microbial/not anti-microbial).	[35]
amp_iamp2l	Prediction of peptides with anti-microbial efficiency (anti-microbial/not anti-microbial).	[36]
amp_modlamp	Prediction of peptides with anti-microbial efficiency (anti-microbial/not anti-microbial).	[37]
atb_antitbp	Prediction of peptides with anti-mycbacterial efficiency (anti-tubercular/not anti-tubercular).	[38]
atb_iantitb	Prediction of peptides with anti-mycbacterial efficiency (anti-tubercular/not anti-tubercular).	[39]
avp_amppred	Prediction of peptides with anti-viral efficiency (anti-viral/not anti-viral).	[27]
avp_avppred	Prediction of peptides with anti-viral efficiency (anti-viral/not anti-viral).	[40]
bce_ibce	Prediction of B-cell epitopes (B-cell epitope/no B-cell epitope).	[41]
cpp_cellppd	Prediction of peptides with penetration capability of cell membranes (cell-penetrating/non cell-penetrating).	[42]
cpp_cellppdmod	Prediction of peptides with penetration capability of cell membranes (cell-penetrating/non cell-penetrating).	[43]
cpp_cpppredfl	Prediction of peptides with penetration capability of cell membranes (cell-penetrating/non cell-penetrating).	[44]
cpp_kelmcpp	Prediction of peptides with penetration capability of cell membranes (cell-penetrating/non cell-penetrating).	[45]
cpp_mixed	Prediction of peptides with penetration capability of cell membranes (cell-penetrating/non cell-penetrating).	[46]

cpp_mlcpp	Prediction of peptides with penetration capability of cell membranes (cell-penetrating/non cell-penetrating).	[47]
cpp_mlcppue	Prediction of uptake efficiency of cell-penetrating peptides (high uptake/low uptake).	[47]
cpp_sanders	Prediction of peptides with penetration capability of cell membranes (cell-penetrating/non cell-penetrating).	[48]
hem_hemopi	Prediction of peptides with hemolytic susceptibility (susceptible/resistant).	[49]
hiv_3tc	Prediction of HIV-1 subtype B drug resistance against Lamivudine (susceptible/resistant).	[50]
hiv_abc	Prediction of HIV-1 subtype B drug resistance against Abacavir (susceptible/resistant).	[50]
hiv_apv	Prediction of HIV-1 subtype B drug resistance against Amprenavir (susceptible/resistant).	[50]
hiv_azt	Prediction of HIV-1 subtype B drug resistance against Zidovudine (susceptible/resistant).	[50]
hiv_bevirimat	Prediction of HIV-1 drug resistance against Bevirimat (susceptible/resistant).	[51]
hiv_d4t	Prediction of HIV-1 subtype B drug resistance against Stavudine (susceptible/resistant).	[50]
hiv_ddi	Prediction of HIV-1 subtype B drug resistance against Didanosin (susceptible/resistant).	[50]
hiv_dlv	Prediction of HIV-1 subtype B drug resistance against Delavirdine (susceptible/resistant).	[50]
hiv_efv	Prediction of HIV-1 subtype B drug resistance against Efavirenz (susceptible/resistant).	[50]
hiv_idv	Prediction of HIV-1 subtype B drug resistance against Indinavir (susceptible/resistant).	[50]
hiv_lpv	Prediction of HIV-1 subtype B drug resistance against Lopinavir (susceptible/resistant).	[50]
hiv_nfv	Prediction of HIV-1 subtype B drug resistance against Nelfinavir (susceptible/resistant).	[50]
hiv_nvp	Prediction of HIV-1 subtype B drug resistance against Nevirapin (susceptible/resistant).	[50]
hiv_protease	Prediction of cleavage by HIV-1 protease (cleavage/no cleavage).	[52]
hiv_rtv	Prediction of HIV-1 subtype B drug resistance against Ritonavir (susceptible/resistant).	[50]
hiv_sqv	Prediction of HIV-1 subtype B drug resistance against Saquinavir (susceptible/resistant).	[50]
hiv_v3	Prediction of HIV-1 V3 loop co-receptor tropism (R5/X4).	[53]
isp_il10pred	Prediction of peptides activating interleukin production (activating/non-activating).	[54]
nep_neuropiprede	Prediction of neuropeptides from insects (neuropeptide/no neuropeptide).	[55]
pip_pipel	Prediction of peptides activating an proinflammatory immune response (activating/non-activating).	[56]
tce_zhao	Prediction of T-cell epitopes (T-cell epitope/no T-cell epitope).	[57]

Supplementary Table 4: Encodings used in this study accompanied by the parameters. The number of parameters is stated in brackets and the total space covered is shown in the adjacent column. Note, that the numbers denote the maximum amount of parameters. However, for particular datasets this number is different, due to the sequence length. Please refer to [2], [58], and Supplementary Note 1 for details on the algorithms. Structure-based encodings are marked with †.

**Group**	**Description**	**Paramters (#)**	**Total param. space**	**Ref.**
aac	Amino Acid Composition	–	1	[58]
aaindex	Amino Acid Index	amino acid index (531)	531	[58]
apaac	Amphiphilic Pseudo-Amino Acid Composition	lambda (30)	30	[58]
asa†	Accessible Solvent Accessibility	–	1	[58]
binary	Binary	–	1	[58]
blomap	Blomap	–	1	[58]
blosum62	Blosum62	–	1	[58]
cgr	Frequency Matrix Chaos Game Representation	Resolution (4), s-factor (2)	8	[50]
cksaagp	Composition of k-Spaced Amino Acid Group Pairs	gap (30)	30	[58]
cksaap	Composition of k-spaced Amino Acid Pairs	gap (30)	30	[58]
ctdc	Composition/ Transition/ Distribution composition	–	1	[58]
ctdd	Composition/ Transition/ Distribution distribution	–	1	[58]
ctdt	Composition/ Transition/ Distribution transition	–	1	[58]
ctriad	Conjoint Triad	–	1	[58]
dde	Dipeptide Deviation from Expected Mean	–	1	[58]
delaunay†	Delaunay triangulation	type (5)	5	[1]
disorder†	Disorder	–	1	[58]
disorderb†	Disorder binary	–	1	[58]
disorderc†	Disorder content	–	1	[58]
distance_distribution†	Distance distribution	–	1	[4]
distance_frequency	Distance frequency	n-terminal (5), c-terminal (5)	25	[6]
dpc	Di-Peptide Composition	–	1	[58]
eaac	Enhanced Amino Acid Composition	window (30)	30	[58]

egaac	Enhanced Grouped Amino Acid Composition	window (8)	8	[58]
electrostatic_hull†	Electrostatic hull	distance (5)	5	[10]
fft	Fast-Fourier-Transform	amino acid index (531)	531	[12]
fldpc	Five Level Di-Peptide Composition	amino acid index (531)	531	[13]
flgc	Five Level Grouping Composition	amino acid index (531)	531	[13]
gaac	Grouped Amino Acid Composition	–	1	[58]
gdpc	Grouped Di-Peptide Composition	–	1	[58]
geary	Geary correlation	n-lag (30)	30	[58]
gtpc	Grouped Tri-Peptide Composition	–	1	[58]
ksctriad	k-Spaced Conjoint Triad	gap (30)	30	[58]
moran	Moran correlation	n-lag (30)	30	[58]
ngram	N-gram	type (6), dim (7)	42	
nmbroto	Normalized Moreau-Broto Autocorrelation	n-lag (30)	30	[58]
paac	Pseudo-Amino Acid Composition	lambda (30)	30	[58]
psekraac	48 pseudo K-tuple reduced amino acids composition	type (19), subtype (2), raac-type (20), k-tuple (3), g-lambda (3)	3420	[58]
qsar†	Quantitative Structure-Activity Relationship	–	1	[16]
qsorder	Quasi-sequence-order	n-lag (30)	30	[58]
socnumber	Sequence-Order-Coupling Number	–	1	[58]
sseb†	Secondary Structure Elements Binary	–	1	[58]
ssec†	Secondary Structure Elements Content	–	1	[58]
ta†	Torsion angle	–	1	[58]
tpc	Tri-Peptide Composition	–	1	[58]
waac	Weighted Amino Acid Composition	amino acid index (531)	531	[13]
zscale	Z-Scale	–	1	[58]

References

[1] Bose, P. and Harrison, R. W. (2011) Encoding protein structure with functions on graphs. *2011 IEEE International Conference on Bioinformatics and Biomedicine Workshops (BIBMW),* pp. 338–344.

[2] Spänig, S. and Heider, D. (2019) Encodings and models for antimicrobial peptide classification for multi-resistant pathogens. *BioData Mining,* **12**(1), 1–29.

[3] Virtanen, P., Gommers, R., Oliphant, T. E., Haberland, M., Reddy, T., Cournapeau, D., Burovski, E., Peterson, P., Weckesser, W., Bright, J., et al. (2020) SciPy 1.0: fundamental algorithms for scientific computing in Python. *Nature Methods,* **17**(3), 261–272.

[4] Sander, O., Sing, T., Sommer, I., Low, A. J., Cheung, P. K., Harrigan, P. R., Lengauer, T., and Domingues, F. S. (2007) Structural descriptors of gp120 V3 loop for the prediction of HIV-1 coreceptor usage. *PLoS Computational Biology,* **3**(3), 0555–0564.

[5] Turlach, B. A. (1993) Bandwidth Selection in Kernel Density Estimation: A Review. *CORE and Institut de Statistique,* **19**, 1–33.

[6] Matsuda, S., Vert, J.-p., Saigo, H., Ueda, N., Toh, H., and Akutsu, T. (2005) A novel representation of protein sequences for prediction of subcellular location using support vector machines. *Protein Science,* **14**, 2804–2813.

[7] Dolinsky, T. J., Czodrowski, P., Li, H., Nielsen, J. E., Jensen, J. H., Klebe, G., and Baker, N. A. (2007) PDB2PQR: Expanding and upgrading automated preparation of biomolecular structures for molecular simulations. *Nucleic Acids Research,* **35**, 522–525.

[8] Baker, N. A., Sept, D., Joseph, S., Holst, M. J., and McCammon, J. A. (2001) Electrostatics of nanosystems: Application to microtubules and the ribosome. *Proc Natl Acad Sci U S A,* **98**(18), 10037–10041.

[9] Heider, D. and Hoffmann, D. (2011) Interpol: An R package for preprocessing of protein sequences. *BioData Mining,* **4**(1), 2–7.

[10] Löchel, H. F., Riemenschneider, M., Frishman, D., and Heider, D. (2018) SCOTCH : Subtype A Coreceptor Tropism Classification in HIV-1. *Bioinformatics,* **34**(15), 2575–2580.

[11] Kawashima, S., Pokarowski, P., Pokarowska, M., Kolinski, A., Katayama, T., and Kanehisa, M. (2008) AAindex: Amino acid index database, progress report 2008. *Nucleic Acids Research,* **36**(SUPPL. 1), 202–205.

[12] Nagarajan, V., Kaushik, N., Murali, B., Zhang, C., Lakhera, S., Elasri, M. O., and Deng, Y. (sep, 2006) A fourier transformation based method to mine peptide space for antimicrobial activity. *BMC Bioinformatics,* **7**(SUPPL.2).

[13] Tantoso, E. and Li, K. B. (2008) AAIndexLoc: Predicting subcellular localization of proteins based on a new representation of sequences using amino acid indices. *Amino Acids,* **35**(2), 345–353.

[14] Wu, C., Berry, M., Shivakumar, S., Mclarty, J., Hunter, L., Searls, D., and Shavlik, J. (1995) Neural Networks for Full-Scale Protein Sequence Classification: Sequence Encoding with Singular Value Decomposition. *Machine Learning,* **21**, 177–193.

[15] Wu, C. H., Whitson, G. M., and Montllor, G. J. (1990) PROCANS: A Protein Classification System Using A Neural Network. *1990 IJCNN International Joint Conference on Neural Networks,* **2**, 91–96.

[16] Haney, E. F., Brito-Sánchez, Y., Trimble, M. J., Mansour, S. C., Cherkasov, A., and Hancock, R. E. (dec, 2018) Computer-aided Discovery of Peptides that Specifically Attack Bacterial Biofilms. *Scientific Reports,* **8**(1).

[17] Moriwaki, H., Tian, Y. S., Kawashita, N., and Takagi, T. (feb, 2018) Mordred: A molecular descriptor calculator. *Journal of Cheminformatics,* **10**(1).

[18] Wiwie, C., Baumbach, J., and Röttger, R. (nov, 2015) Comparing the performance of biomedical clustering methods. *Nature Methods,* **12**(11), 1033–1038.

[19] Davies, D. L. and Bouldin, D. W. (1979) A Cluster Separation Measure. *IEEE Transactions on Pattern Analysis and Machine Intelligence,* **PAMI-1**(2), 224–227.

[20] Van Der Maaten, L. and Hinton, G. (2008) Visualizing Data using t-SNE. *Journal of Machine Learning Research,* **9**, 2579–2605.

[21] Schubert, E. and Rousseeuw, P. (2019) Faster k-Medoids Clustering: Improving the PAM, CLARA, and CLARANS Algorithms. *Amato G., Gennaro C., Oria V., Radovanović M. (eds) Similarity Search and Applications. SISAP 2019. Lecture Notes in Computer Science,* **11807**.

[22] Wold, S., Esbensen, K., and Geladi, P. (1987) Principal Component Analysis. *Chemometrics and Intelligent Laboratory Systems,* **2**, 37–52.

[23] Nagpal, G., Chaudhary, K., Agrawal, P., and Raghava, G. P. (jul, 2018) Computer-aided prediction of antigen presenting cell modulators for designing peptide-based vaccine adjuvants. *Journal of Translational Medicine,* **16**(1).

[24] Tyagi, A., Kapoor, P., Kumar, R., Chaudhary, K., Gautam, A., and Raghava, G. P. (2013) In silico models for designing and discovering novel anticancer peptides. *Scientific Reports,* **3**.

[25] Chen, W., Ding, H., Feng, P., Lin, H., and Chou, K.-C. (2016) iACP: a sequence-based tool for identifying anticancer peptides. *Oncotarget,* **7**(13).

[26] Manavalan, B., Basith, S., Shin, T. H., Choi, S., Kim, M. O., and Lee, G. (2017) MLACP: machine-learning-based prediction of anticancer peptides. *Oncotarget,* **8**(44), 77121–77136.

[27] Meher, P. K., Sahu, T. K., Saini, V., and Rao, A. R. (feb, 2017) Predicting antimicrobial peptides with improved accuracy by incorporating the compositional, physico-chemical and structural features into Chou's general PseAAC. *Scientific Reports,* **7**.

[28] Agrawal, P., Bhalla, S., Chaudhary, K., Kumar, R., Sharma, M., and Raghava, G. P. (feb, 2018) In silico approach for prediction of antifungal peptides. *Frontiers in Microbiology,* **9**(FEB).

[29] Manavalan, B., Govindaraj, R. G., Shin, T. H., Kim, M. O., and Lee, G. (2018) iBCE-EL: A New Ensemble Learning Framework for Improved Linear B-Cell Epitope Prediction. *Frontiers in immunology,* **9**, 1695.

[30] Gupta, S., Sharma, A. K., Shastri, V., Madhu, M. K., and Sharma, V. K. (jan, 2017) Prediction of anti-inflammatory proteins/peptides: An insilico approach. *Journal of Translational Medicine,* **15**(1).

[31] Lata, S., Sharma, B. K., and Raghava, G. P. (jul, 2007) Analysis and prediction of antibacterial peptides. *BMC Bioinformatics,* **8**.

[32] Su, X., Xu, J., Yin, Y., Quan, X., and Zhang, H. (dec, 2019) Antimicrobial peptide identification using multi-scale convolutional network. *BMC Bioinformatics,* **20**(1).

[33] Porto, W. F., Pires, Á. S., and Franco, O. L. (dec, 2012) CS-AMPPred: An Updated SVM Model for Antimicrobial Activity Prediction in Cysteine-Stabilized Peptides. *PLoS ONE,* **7**(12).

[34] Fernandes, F. C., Rigden, D. J., and Franco, O. L. (2012) Prediction of antimicrobial peptides based on the adaptive neuro-fuzzy inference system application.. *Biopolymers,* **98**(4), 280–287.

[35] Polanco González, C., Nuño Maganda, M. A., Arias-Estrada, M., and del Rio, G. (2011) An FPGA implementation to detect selective cationic antibacterial peptides. *PLoS ONE,* **6**(6).

[36] Xiao, X., Wang, P., Lin, W. Z., Jia, J. H., and Chou, K. C. (may, 2013) IAMP-2L: A two-level multi-label classifier for identifying antimicrobial peptides and their functional types. *Analytical Biochemistry,* **436**(2), 168–177.

[37] Müller, A. T., Gabernet, G., Hiss, J. A., and Schneider, G. (2017) modlAMP: Python for antimicrobial peptides. *Bioinformatics,* **33**(17), 2753–2755.

[38] Usmani, S. S., Bhalla, S., and Raghava, G. P. (aug, 2018) Prediction of antitubercular peptides from sequence information using ensemble classifier and hybrid features. *Frontiers in Pharmacology,* **9**(AUG).

[39] Khatun, S., Hasan, M., and Kurata, H. (nov, 2019) Efficient computational model for identification of antitubercular peptides by integrating amino acid patterns and properties. *FEBS Letters,* **593**(21), 3029–3039.

[40] Thakur, N., Qureshi, A., and Kumar, M. (jul, 2012) AVPpred: Collection and prediction of highly effective antiviral peptides. *Nucleic Acids Research,* **40**(W1).

[41] Manavalan, B., Govindaraj, R. G., Shin, T. H., Kim, M. O., and Lee, G. (2018) iBCE-EL: A New Ensemble Learning Framework for Improved Linear B-Cell Epitope Prediction. *Frontiers in immunology,* **9**, 1695.

[42] Gautam, A., Chaudhary, K., Kumar, R., Sharma, A., Kapoor, P., Tyagi, A., and Raghava, G. P. (mar, 2013) In silico approaches for designing highly effective cell penetrating peptides. *Journal of Translational Medicine,* **11**(1).

[43] Kumar, V., Agrawal, P., Kumar, R., Bhalla, S., Usmani, S. S., Varshney, G. C., and Raghava, G. P. (apr, 2018) Prediction of cell-penetrating potential of modified peptides containing natural and chemically modified residues. *Frontiers in Microbiology,* **9**(APR).

[44] Qiang, X., Zhou, C., Ye, X., Du, P. F., Su, R., and Wei, L. (sep, 2018) CPPred-FL: A sequence-based predictor for large-scale identification of cell-penetrating peptides by feature representation learning. *Briefings in Bioinformatics,* **21**(1), 11–23.

[45] Pandey, P., Patel, V., George, N. V., and Mallajosyula, S. S. (sep, 2018) KELM-CPPpred: Kernel Extreme Learning Machine Based Prediction Model for Cell-Penetrating Peptides. *Journal of Proteome Research,* **17**(9), 3214–3222.

[46] Dobchev, D. A., Mäger, I., Tulp, I., Karelson, G., Tamm, T., Tämm, K., Jänes, J., Langel, Ü., and Karelson, M. (2010) Prediction of Cell-Penetrating Peptides Using Artificial Neural Networks. *Current Computer-Aided Drug Design,* **6**, 79–89.

[47] Manavalan, B., Subramaniyam, S., Shin, T. H., Kim, M. O., and Lee, G. (aug, 2018) Machine-Learning-Based Prediction of Cell-Penetrating Peptides and Their Uptake Efficiency with Improved Accuracy. *Journal of Proteome Research,* **17**(8), 2715–2726.

[48] Sanders, W. S., Johnston, C. I., Bridges, S. M., Burgess, S. C., and Willeford, K. O. (jul, 2011) Prediction of Cell Penetrating Peptides by Support Vector Machines. *PLoS Computational Biology,* **7**(7).

[49] Chaudhary, K., Kumar, R., Singh, S., Tuknait, A., Gautam, A., Mathur, D., Anand, P., Varshney, G. C., and Raghava, G. P. (mar, 2016) A web server and mobile app for computing hemolytic potency of peptides. *Scientific Reports,* **6**.

[50] Löchel, H. F., Eger, D., Sperlea, T., and Heider, D. (2020) Deep learning on chaos game representation for proteins. *Bioinformatics (Oxford, England),* **36**(1), 272–279.

[51] Heider, D., Verheyen, J., and Hoffmann, D. (2010) Predicting Bevirimat resistance of HIV-1 from genotype. *BMC Bioinformatics,* **11**(37), 1–9.

[52] Rögnvaldsson, T., You, L., and Garwicz, D. (apr, 2015) State of the art prediction of HIV-1 protease cleavage sites. *Bioinformatics,* **31**(8), 1204–1210.

[53] Dybowski, J. N., Heider, D., and Hoffmann, D. (apr, 2010) Prediction of co-receptor usage of HIV-1 from genotype. *PLoS Computational Biology,* **6**(4).

[54] Nagpal, G., Usmani, S. S., Dhanda, S. K., Kaur, H., Singh, S., Sharma, M., and Raghava, G. P. (feb, 2017) Computer-aided designing of immunosuppressive peptides based on IL-10 inducing potential. *Scientific Reports,* **7**.

[55] Agrawal, P., Kumar, S., Singh, A., Raghava, G. P., and Singh, I. K. (dec, 2019) NeuroPIpred: a tool to predict, design and scan insect neuropeptides. *Scientific Reports,* **9**(1).

[56] Manavalan, B., Shin, T. H., Kim, M. O., and Lee, G. (2018) PIP-EL: A New Ensemble Learning Method for Improved Proinflammatory Peptide Predictions. *Frontiers in immunology,* **9**, 1783.

[57] Zhao, Y., Pinilla, C., Valmori, D., Martin, R., and Simon, R. (oct, 2003) Application of support vector machines for T-cell epitopes prediction. *Bioinformatics,* **19**(15), 1978–1984.

[58] Chen, Z., Zhao, P., Li, F., Leier, A., Marquez-Lago, T. T., Wang, Y., Webb, G. I., Smith, A. I., Daly, R. J., Chou, K. C., et al. (2018) IFeature: A Python package and web server for features extraction and selection from protein and peptide sequences. *Bioinformatics,* **34**(14), 2499–2502.
